# Amyloid-β (Aβ) immunotherapy induced microhemorrhages are associated with activated perivascular macrophages and peripheral monocyte recruitment in Alzheimer’s disease mice

**DOI:** 10.1186/s13024-023-00649-w

**Published:** 2023-08-30

**Authors:** Xavier Taylor, Isaiah M. Clark, Griffin J. Fitzgerald, Herold Oluoch, Justin T. Hole, Ronald B. DeMattos, Yaming Wang, Feng Pan

**Affiliations:** Neuroscience Discovery, Lilly Research Laboratories, Eli Lilly and Company, Indianapolis, IN 46285 USA

## Abstract

**Background:**

Amyloid-related imaging abnormalities (ARIA) have been identified as the most common and serious adverse events resulting from pathological changes in the cerebral vasculature during several recent anti-amyloid-β (Aβ) immunotherapy trials. However, the precise cellular and molecular mechanisms underlying how amyloid immunotherapy enhances cerebral amyloid angiopathy (CAA)-mediated alterations in vascular permeability and microhemorrhages are not currently understood. Interestingly, brain perivascular macrophages have been implicated in regulating CAA deposition and cerebrovascular function however, further investigations are required to understand how perivascular macrophages play a role in enhancing CAA-related vascular permeability and microhemorrhages associated with amyloid immunotherapy.

**Methods:**

In this study, we examined immune responses induced by amyloid-targeting antibodies and CAA-induced microhemorrhages using histology and gene expression analyses in Alzheimer’s disease (AD) mouse models and primary culture systems.

**Results:**

In the present study, we demonstrate that anti-Aβ (3D6) immunotherapy leads to the formation of an antibody immune complex with vascular amyloid deposits and induces the activation of CD169^+^ perivascular macrophages. We show that macrophages activated by antibody mediated Fc receptor signaling have increased expression of inflammatory signaling and extracellular matrix remodeling genes such as Timp1 and MMP9 in vitro and confirm these key findings in vivo. Finally, we demonstrate enhanced vascular permeability of plasma proteins and recruitment of inflammatory monocytes around vascular amyloid deposits, which are associated with hemosiderin deposits from cerebral microhemorrhages, suggesting the multidimensional roles of activated perivascular macrophages in response to Aβ immunotherapy.

**Conclusions:**

In summary, our study establishes a connection between Aβ antibodies engaged at CAA deposits, the activation of perivascular macrophages, and the upregulation of genes involved in vascular permeability. However, the implications of this phenomenon on the susceptibility to microhemorrhages remain to be fully elucidated. Further investigations are warranted to determine the precise role of CD169 + perivascular macrophages in enhancing CAA-mediated vascular permeability, extravasation of plasma proteins, and infiltration of immune cells associated with microhemorrhages.

**Supplementary Information:**

The online version contains supplementary material available at 10.1186/s13024-023-00649-w.

## Introduction

Anti-amyloid immunotherapy has been tested in multiple late-stage clinical trials in which treated patients demonstrated significant improvements in soluble biomarkers, amyloid pathology, and cognitive functions, making it the most advanced disease-modifying therapeutic option [1, 2]. The general hypothesis is that dosing of anti-amyloid-β (Aβ) antibodies will drive immune responses in the central nervous system (CNS) to neutralize aggregated Aβ by facilitating the clearance and removal of amyloid. The enthusiasm for these immunotherapies is often hindered by common and sometimes severe adverse events, amyloid-related imaging abnormalities (ARIA), observed in several clinical trials [3–5]. Multiple lines of evidence indicate that ARIA is linked to vascular amyloid deposition, also known as cerebral amyloid angiopathy (CAA), suggesting CAA as a key neuropathological component associated with treatment-related ARIA [6]. However, the mechanisms underlying how amyloid immunotherapy enhances CAA-mediated vascular permeability and microhemorrhages are not currently understood.

Microglia residing in the brain parenchyma have been extensively studied in neurodegeneration, while meningeal, parenchymal border, and circulating immune cells residing in the perivascular and leptomeningeal spaces of blood vessels are largely underexplored. Recent studies have identified several distinctive subsets of immune cells located in specific anatomical locations that participate in a broad spectrum of processes under normal and pathological conditions [7, 8]. During normal vascular development in the CNS, perivascular macrophages directly interact with the developing vasculature, influencing each phase of the angiogenic process to facilitate the sprouting [9], branching [10] and anastomosis [11] of blood vessels. Additionally, perivascular macrophages have been shown to protect against bacterial meningitis [12] and to aid in the clearance of vascular Aβ deposition in mouse models of AD [13–16]. However, they can also mediate oxidative stress and cerebrovascular dysfunction induced by Aβ peptides [17]. Importantly, neuropathological findings in a patient receiving Aβ immunotherapy have suggested the involvement of perivascular macrophages in Aβ phagocytosis and clearance within cerebral blood vessels, emphasizing their role in the host response to Aβ immunotherapy [18]. While the precise role of perivascular macrophages in ARIA induction remains unknown, the occurrence of microhemorrhages has been observed within perivascular compartments of the brain, regardless of whether the immunotherapy is active [19] or passive [20–23] suggesting the potential involvement of perivascular macrophages in responding to immune complexes formed by vascular amyloid-targeting antibodies. Therefore, utilizing a well-established mouse model of Aβ immunotherapy-induced microhemorrhages, we aimed to determine the immune cell populations associated with CAA-mediated vascular permeability, plasma protein extravasation, and hemorrhagic resolution.

Herein, our results demonstrate that Aβ immunotherapy in AD mice strongly induces the activation of perivascular macrophages and its colocalization with CAA antibody immune complexes. Furthermore, we determined in vitro cultured macrophages activated by antibody mediated Fc receptor signaling have a significant increase in the expression of inflammatory signaling and extracellular matrix genes such as Timp1 and MMP9 and confirm these key findings in vivo. Finally, we demonstrate that inflammatory monocytes are highly abundant around vascular amyloid deposits and are associated with hemosiderin deposits of cerebral microhemorrhages induced by Aβ immunotherapy.

## Results

### Aβ immunotherapy provokes CD169^+^ perivascular macrophages association with vascular amyloid deposits

**Fig. 1:**
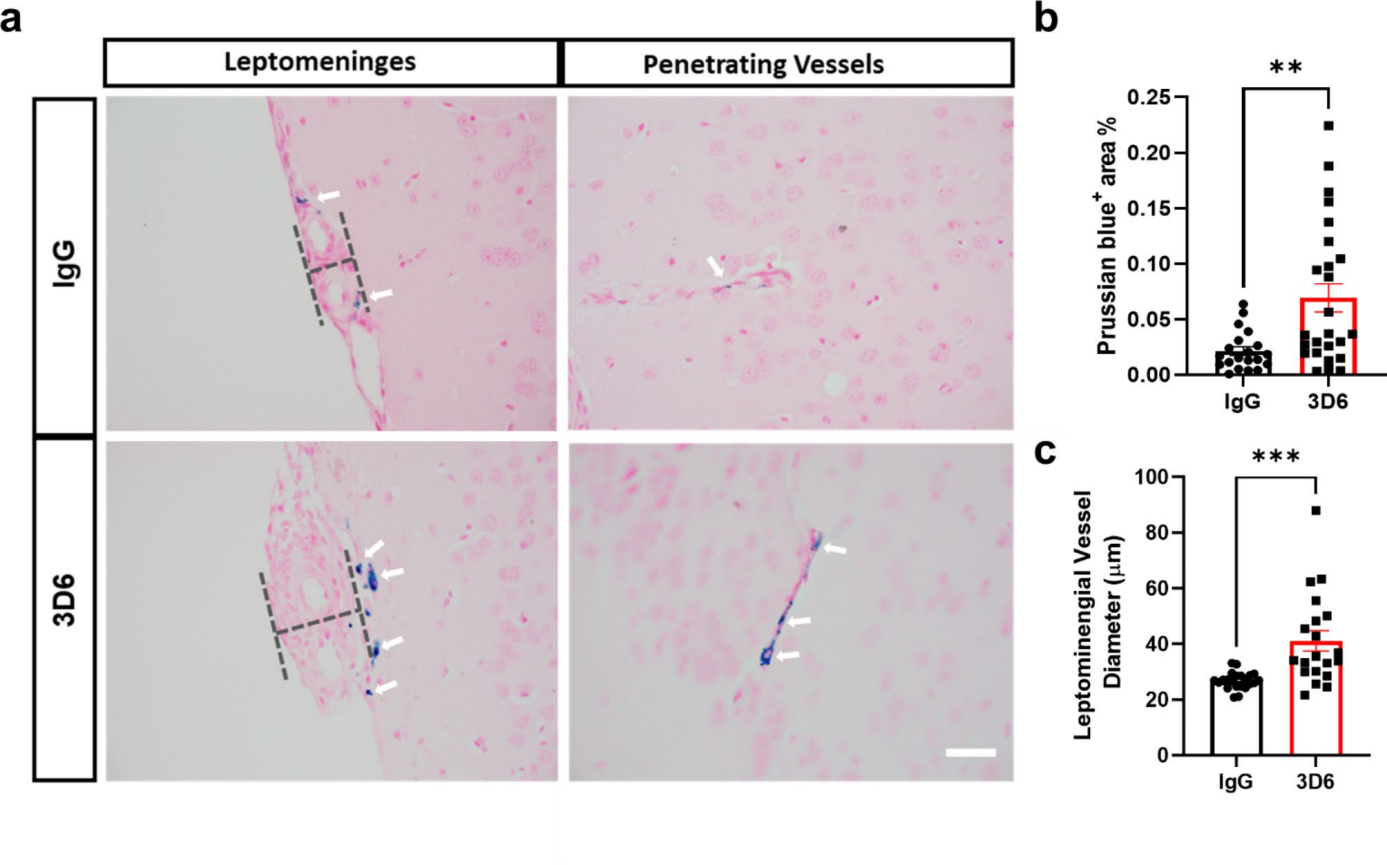
3D6 treatment induces microhemorrhages in PDAPP mice. **(a)** Prussian Blue (hemosiderin, blue) labeled microhemorrhages in the leptomeninges and penetrating vessels of PDAPP mice treated with IgG or 3D6. **(b)** Quantification of Prussian blue + area (%) in brain coronal sections of IgG or 3D6-treated mice. **(c)** Measurement of the diameter (dashed line) of the leptomeninges in mice treated with IgG or 3D6. For quantifications, total coronal sections were used, and each data point indicates an animal n = 20–25 (mice). All are representative images of 26-month-old PDAPP mice. Results are shown as ± SEM, asterisks indicate significant differences, where **p < 0.01 and ***p < 0.001 by unpaired Student’s t test. Scale bar 40 μm

**Fig. 2:**
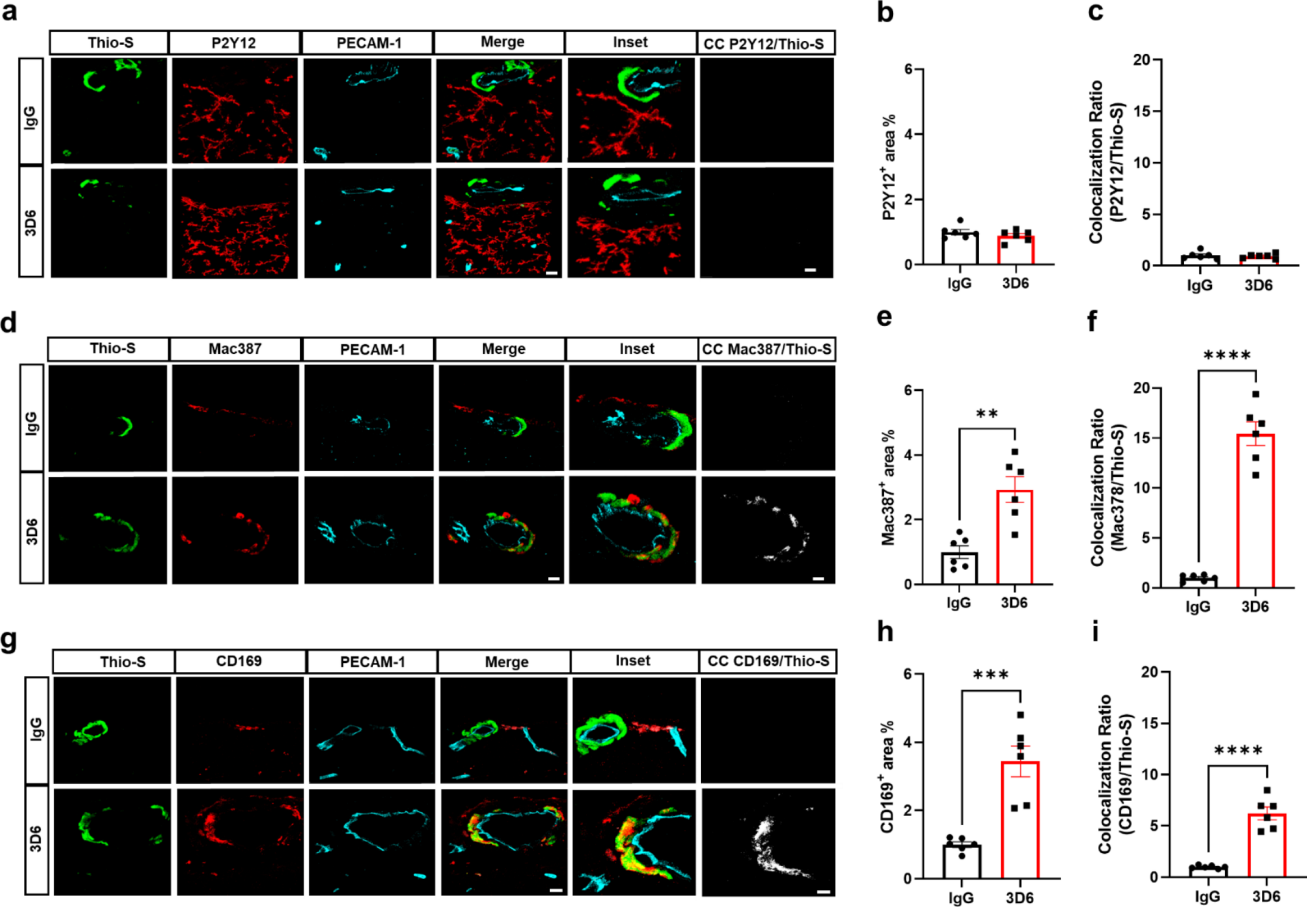
Perivascular macrophages are highly associated with vascular amyloid in 3D6 treated PDAPP Mice. **(a)** Triple immunofluorescence of amyloid (Thio-S, green), microglia (P2Y12, red) and endothelial cells (PECAM-1, cyan) in PDAPP mice treated with 3D6 or IgG control. Thio-S, P2Y12 and PECAM-1 immunoreactivity overlay (Merge). Colocalization analysis (white). **(b)** Quantification of P2Y12^+^ area (%) of IgG or 3D6 treated mice. **(c)** Quantification of colocalization ratio of Thio-S and P2Y12. **(d)** Triple immunofluorescence of amyloid (Thio-S, green), macrophages (Mac387, red) and endothelial cells (PECAM-1, cyan) in PDAPP mice treated with 3D6 or IgG control. Thio-S, Mac387 and PECAM-1 immunoreactivity overlay (Merge). Colocalization analysis (white). **(e)** Quantification of Mac387^+^ area (%) of IgG or 3D6 treated mice. **(f)** Quantification of colocalization ratio of Thio-S and Mac387. **(g)** Triple immunofluorescence of amyloid (Thio-S, green), perivascular macrophages (CD169, red) and endothelial cells (PECAM-1, cyan) in PDAPP mice treated with 3D6 or IgG control. Thio-S, CD169 and PECAM-1 immunoreactivity overlay (Merge). Colocalization analysis (white). **(h)** Quantification of CD169^+^ area (%) of IgG or 3D6 treated mice. **(i)** Quantification of colocalization ratio of Thio-S and CD169. The number of vascular amyloid deposits analyzed was 8–10 per animal. Results are shown as ± SEM of n = 6 (mice). Asterisks indicate significant differences, where **p < 0.01, *** p < 0.001 or ****p < 0.0001 by unpaired Student’s t test. Scale bar 5 μm CC or 10 μm merge, respectively

**Fig. 3:**
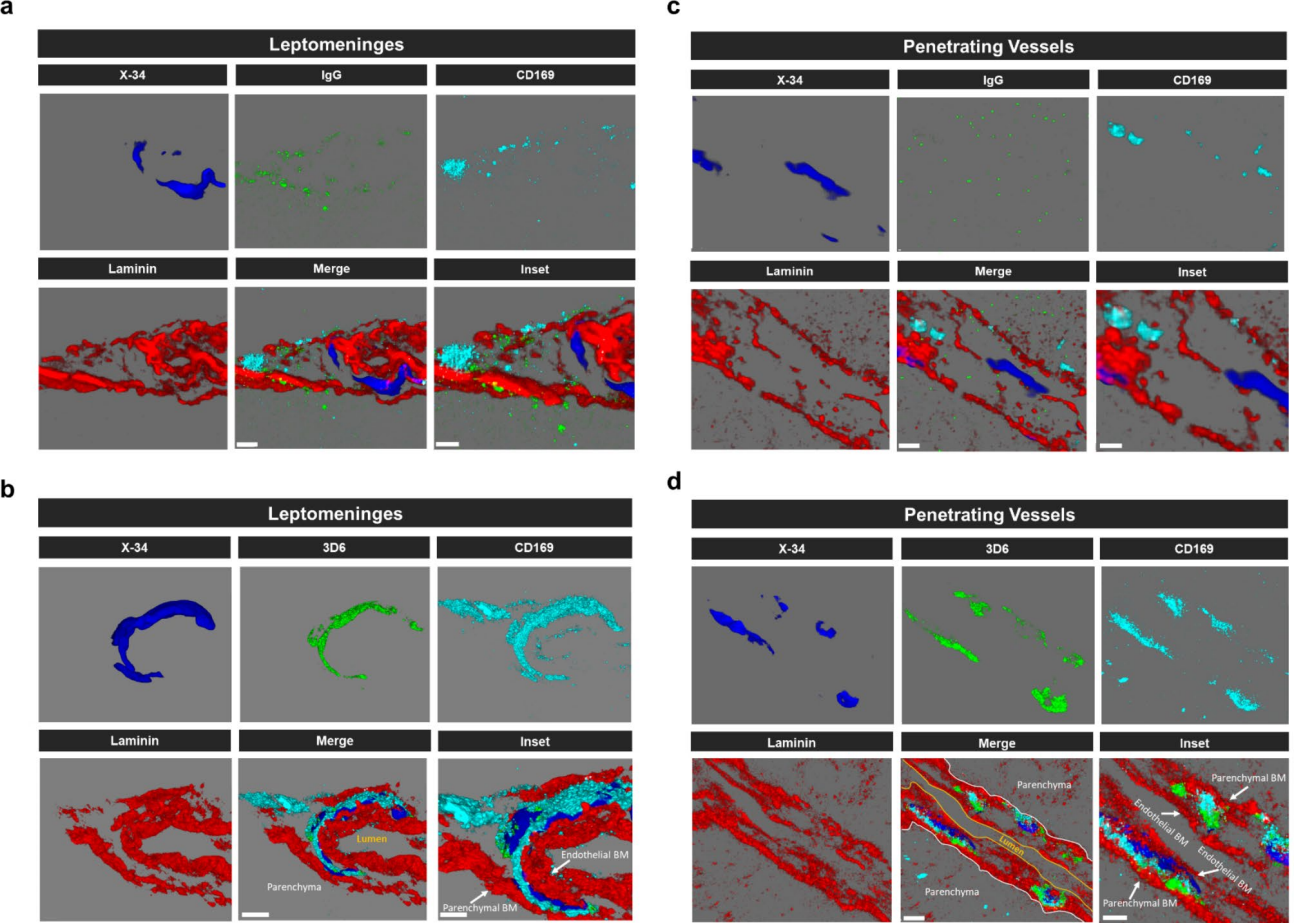
Engagement of CD169^+^ perivascular macrophages with vascular amyloid immune complex in 3D6 treated hTau APP KI Mice. **(a-d)** Four-color immunofluorescence of amyloid (X-34, blue), antibody (IgG or 3D6, green), laminin (red) and perivascular macrophage (CD169, cyan) in hTau APP KI mice treated with biotinylated 3D6 or IgG control. X-34, antibody, laminin and CD169 immunoreactivity overlay (Merge). Scale bar 3 μm merge or 2 μm inset, respectively

It has been well established that anti-Aβ immunotherapy using Bapineuzumab and the murine monoclonal antibody equivalent 3D6 reduces total Aβ levels however, its administration also elicits notable inflammatory response, intensifies vascular permeability, compromises vascular integrity, and triggers the occurrences of microhemorrhages or ARIA in mouse models and AD patients [24–26]. PDAPP mice exhibit distinctive features of vascular amyloid deposition, primarily concentrated within the leptomeninges, but also penetrating vessels of the brain and display significant microhemorrhages in response to anti-Aβ (3D6) immunotherapy [26]. Therefore, we aimed to determine anti-Aβ immunotherapy mediated immune cell interactions around the sites of vascular amyloid accumulation, plasma protein extravasation, and microhemorrhages in 23-month old PDAPP animals. To validate the efficacy of Aβ immunotherapy in reducing Aβ immunoreactivity, PDAPP mice were subjected to a three-month treatment with either 3D6 or IgG control antibody. Subsequent staining of coronal sections confirmed a reduction in 3D6 immunoreactivity, encompassing both parenchymal and vascular Aβ deposits (Supplemental Fig. 1, a-d). To detect hemorrhagic events, sections were stained with Perl’s Prussian blue dye. Our findings revealed a significant increase in the Prussian blue^+^ area, indicating a higher incidence of hemorrhages and an enlargement of leptomeningeal vessel diameter in mice treated with 3D6 compared to the control IgG group (Fig. 1, a-c). These observations align with the documented increase in ARIA following Aβ immunotherapy observed in clinical trials [24, 25]. Considering the appreciable variability in CAA among PDAPP mice and our previous findings demonstrating 3D6-induced exacerbation of microhemorrhage can occur even without plaque reduction [26], a subset of 6 animals from the total microhemorrhage groups were blindly selected for further investigations and subgroup analysis. To determine immune cell interactions following Aβ immunotherapy, we evaluated microglia and macrophages associations with vascular amyloid deposits. We performed triple staining with Thio-S, PECAM-1, and P2Y12 to determine microglia association with vascular amyloid accumulation in the leptomeninges. Interestingly, we found no significant differences in P2Y12^+^ area or colocalization ratio relative to control IgG (Fig. 2, a-c). The absence of microglia associated with vascular amyloid was confirmed in penetrating vessels of the cortex with no significant differences in P2Y12^+^ area or colocalization ratio relative to control IgG (Supplemental Fig. 2, a-c), supporting previous reports [27, 28]. Additionally, Clec7a a well-known marker for plaque-associated microglia confirms reactive microglia are exclusively associated with parenchymal amyloid plaques not vascular amyloid deposits (Supplemental Fig. 3, a-b). To determine macrophages’ association to vascular amyloid accumulation, we performed triple staining with Thio-S, PECAM-1, and Mac387, an intracytoplasmic antigen found in macrophages but not in microglia [29–31]. Our results show macrophages are highly associated with vascular amyloid in 3D6 treated PDAPP mice with significant increases in Mac387^+^ area and colocalization ratio relative to control IgG (Fig. 2, d-f). To confirm these results, we evaluated macrophages’ association with vascular amyloid deposits in penetrating vessels of the cortex and observed significant increases in Mac387^+^ area and colocalization ratio relative to control IgG (Supplemental Fig. 2, d-f). Next, we sought to determine the subpopulation of macrophages associated to vascular amyloid deposits after Aβ immunotherapy. Previously, a subpopulation of perivascular macrophages has been identified that express CD169 (Sialic acid-binding immunoglobulin-type lectins-1, Siglec1) molecules on their surface and exhibit a unique anatomical location and functional phenotype in secondary lymphoid organs at the interface between circulating fluids where blood and lymph enter and leave [32]. Surprisingly, triple staining with Thio-S, PECAM-1, and CD169 revealed that CD169^+^ perivascular macrophages are highly associated with vascular amyloid in 3D6 treated PDAPP mice, with significant increases in CD169^+^ area and colocalization ratio compared to control IgG (Fig. 2, g-i). To confirm these results, we evaluated CD169^+^ perivascular macrophages’ association with vascular amyloid deposits in penetrating vessels of the cortex and observed significant increases in CD169^+^ area and colocalization ratio relative to control IgG (Supplemental Fig. 2, g-i). Notably, significant increases in CD169^+^ area were only observed with vascular amyloid^+^ vessels in 3D6 treated PDAPP suggesting the potential engagement of perivascular macrophage with vascular amyloid immune complexes (Supplemental Fig. 4, a-d). To further investigate the association of perivascular macrophages with vascular amyloid immune complexes, we employed an additional mouse model of Alzheimer’s Disease. Specifically, 24-month-old hTau APP KI mice were treated with biotinylated-3D6 or IgG for a duration of 1 month, followed by four-color immunofluorescence staining with X-34, streptavidin, CD169, and laminin—a marker for basement membranes. Our results confirm 3D6-Aβ immunotherapy promotes the formation of an antibody immune complex with vascular amyloid and is associated with CD169^+^ perivascular macrophages in the leptomeninges and penetrating vessels of the cortex in hTau APP KI mice (Fig. 3, a-d and Movies 1–4). Furthermore, we performed triple immunofluorescence staining with X-34, CD206 and CD163 both widely accepted markers for perivascular macrophages but not microglia [33], demonstrating their association with vascular amyloid after 3D6-Aβ immunotherapy (Supplemental Fig. 5). These findings indicate 3D6-Aβ immunotherapy results in increased occurrences of microhemorrhages, altered cerebrovascular structure and the formation of an antibody immune complex with vascular amyloid deposits associated with perivascular macrophage in the leptomeninges and penetrating vessels of the cortex in mouse models of AD.

### Macrophages activated by antibody mediated fc receptor signaling are enriched in inflammatory and extracellular matrix remodeling genes

**Fig. 4:**
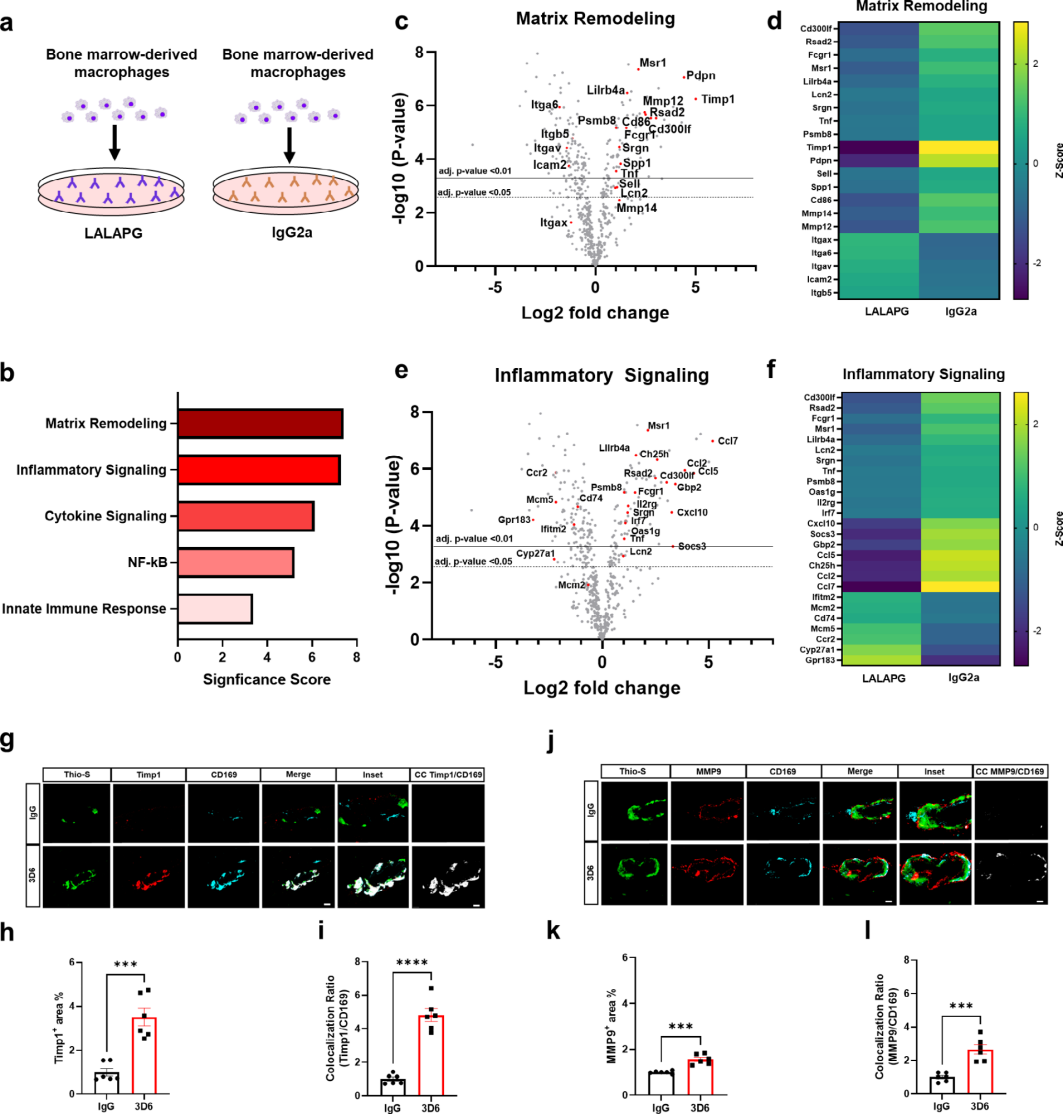
Aβ immunotherapy activated perivascular macrophages are enriched in inflammatory and extracellular matrix remodeling genes. **(a)** Schematic diagram showing macrophages activated by IgG2a or LALAPG immunodeficient control antibodies. **(b)** NanoString gene set enrichment analysis significance scores of macrophages activated IgG2a vs. control LALAPG. **(c)** Volcano plots showing directed differentially expressed matrix remodeling genes in IgG2a activated macrophages vs. control LALAPG activated macrophages. **(d)** NanoString generated heatmap of matrix remodeling gene expression (yellow = upregulation, purple = downregulation). **(e)** Volcano plots showing directed differentially expressed inflammatory signaling genes in IgG2a activated macrophages vs. control LALAPG activated macrophages. **(f)** NanoString generated heatmap of inflammatory signaling gene expression (yellow = upregulation, purple = downregulation). **(g)** Triple immunofluorescence of amyloid (Thio-S, green), Timp1 (red) and perivascular macrophages (CD169, cyan) in PDAPP mice treated with 3D6 or IgG control. Thio-S, Timp1 and CD169 immunoreactivity overlay (Merge). Colocalization analysis (white). **(h)** Quantification of Timp1^+^ area (%) of IgG or 3D6 treated mice. **(i)** Quantification of colocalization ratio of Timp1 and CD169. **(j)** Triple immunofluorescence of amyloid (Thio-S, green), MMP9 (red) and perivascular macrophages (CD169, cyan) in PDAPP mice treated with 3D6 or IgG control. Thio-S, MMP9 and CD169 immunoreactivity overlay (Merge). Colocalization analysis (white). **(k)** Quantification of MMP9^+^ area (%) of IgG or 3D6 treated mice. **(l)** Quantification of colocalization ratio of MMP9 and CD169. The number of vascular amyloid deposits analyzed was 8–10 per animal. Results are shown as ± SEM of n = 6 (mice). Asterisks indicate significant differences, where *** p < 0.001 and ****p < 0.0001 by unpaired Student’s t test. Horizontal line values correspond to adj. p-value < 0.01(solid), adj. p-value < 0.05(dashed). Scale bar 5 μm CC or 10 μm merge, respectively

Given the identified association of CD169^+^ perivascular macrophages to vascular amyloid deposits after Aβ immunotherapy, we wondered whether antibody-immune cell interactions would generate unique macrophage gene expression profiles. To investigate the broader mechanisms of immune activation in macrophages, we utilized primary bone marrow-derived macrophages (BMDM) as a well-established and commonly employed in vitro model for studying the polarization of activated macrophages [34]. To examine the effects of Fc receptor activation, we cultured and plated BMDM in wells coated with either high Fc receptor activating antibody IgG2a or an isotype control antibody harboring the LALAPG mutation, which selectively eliminates Fc-FcR mediated effector functions while preserving antibody affinity [35] (Fig. 4a). Next, we performed a partial transcriptomic analysis using NanoString Technologies’ neuroinflammation panel that evaluates the expression of 770 genes, allowing us to perform gene set enrichment analysis (GSEA). GSEA revealed that BMDM activated by Fc receptor signaling have gene sets and pathways enriched in extracellular matrix remodeling and inflammatory signaling (Fig. 4b, Supplementary Data 1). Importantly, volcano plots and heatmaps of directed differentially expressed genes show that matrix remodeling genes corresponding to matrix metalloproteinase (MMPs) and tissue inhibitors of metalloproteinase (TIMPs) were the most significantly differentially expressed, indicating dysregulated tissue remodeling and homeostasis (Fig. 4, c and d, Supplementary Data 2). Furthermore, volcano plots and heatmaps of directed differentially expressed genes show inflammatory signaling genes corresponding to CCL7, CCL5 and CCL2 were the most significantly differentially expressed, suggesting dysregulated chemotaxis and inflammatory responses (Fig. 4, e and f, Supplementary Data 2). Additionally, markers Pdpn, Timp1 and Msr1 were subsequently validated by qPCR confirming their enrichment in inflammatory and extracellular matrix remodeling pathways (Supplemental Fig. 6, a-c). To validate that LALAPG isotope control antibody ablates Fc receptor signaling, we cultured and plated primary BMDM in wells coated with LALPG or untreated BMDM. Volcano plots of directed differentially expressed genes verify that no genes met the criteria of adjusted p-value cutoff < 0.05 and were not significantly differentially expressed (Supplemental Fig. 7, Supplementary Data 2).

Through NanoString analysis of genes upregulated in BMDM activated by Fc receptor signaling, we identified Timp1 as an extracellular matrix gene of particular interest, with known roles in inhibiting MMPs, regulating blood-brain barrier integrity and biological activities independent of metalloproteinases directly or indirectly affecting wound healing and inflammation [36]. To determine if Timp1 can be used as a molecular signature of CD169^+^ perivascular macrophages associated with vascular amyloid after Aβ immunotherapy, we performed triple staining with Thio-S, CD169, and Timp1. Remarkably, our results show significant increases in Timp1^+^ area and colocalization ratio with CD169^+^ perivascular macrophages relative to control IgG (Fig. 4, g-i). To confirm these results, we evaluated penetrating vessels of the cortex and observed significant increases in Timp1^+^ area and colocalization ratio with CD169^+^ perivascular macrophages relative to control IgG (Supplemental Fig. 8, a-c), supporting our NanoString results (Fig. 4, c and d, GEO: GSE222466). Notably Timp1^+^ immunoreactivity was not detected in astrocytes, microglia or endothelial cells affirming its association with activated perivascular macrophages (Supplemental Fig. 9, a-b). Interestingly, Timp1 is a MMP9-specific tissue inhibitor, binding both pro-MMP-9 and mature MMP-9, and elevations in Timp1 has been proposed to be a protective response to ischemic insult to combat increased levels of MMP-9 [37, 38]. To identify if CD169^+^ perivascular macrophages were also MMP9^+^, we performed triple staining with Thio-S, CD169 and MMP9 and observed significant increases in MMP9^+^ area and colocalization ratio with CD169^+^ perivascular macrophages relative to control IgG (Fig. 4, j-l). To confirm these results, we evaluated penetrating vessels of the cortex and observed significant increases in MMP9^+^ area and colocalization ratio with CD169^+^ perivascular macrophages relative to control IgG (Supplemental Fig. 8, d-f).

It is well known that anti-Aβ immunotherapy induces activation of MMPs that may proteolyze cerebrovascular basement membranes and tight junction proteins, which could compromise vascular integrity, resulting in barrier leakage and extravasation [39, 40]. To determine blood brain barrier (BBB) leakage and extravasation of plasma proteins, we performed triple staining with Thio-S, PECAM-1, and Fibrinogen, a coagulation protein absent in the brain, unless there is a breach of cerebral vasculature blood-brain or permeability barrier [41–43]. Our results show fibrinogen is increased around vascular amyloid deposits in 3D6 treated PDAPP mice with significant increases in fibrinogen^+^ area in the leptomeninges and in penetrating vessels of the cortex (Supplemental Fig. 10, a-d) relative to control IgG. These observations demonstrate that Aβ immunotherapy is associated with induction of Timp1^+^ and MMP9^+^ perivascular macrophages, basement membrane remodeling, and increased fibrinogen extravasation associated with vascular amyloid deposits. However, further studies will be necessary to determine the extent to which cerebrovascular targeting amyloid immunotherapy enhances the MMP9/Timp1 ratio in influencing BBB permeability, leakage, and extravasation of blood plasma protein.

### Inflammatory monocytes are highly abundant around vascular amyloid deposits and associated with Aβ immunotherapy induced microhemorrhages

**Fig. 5:**
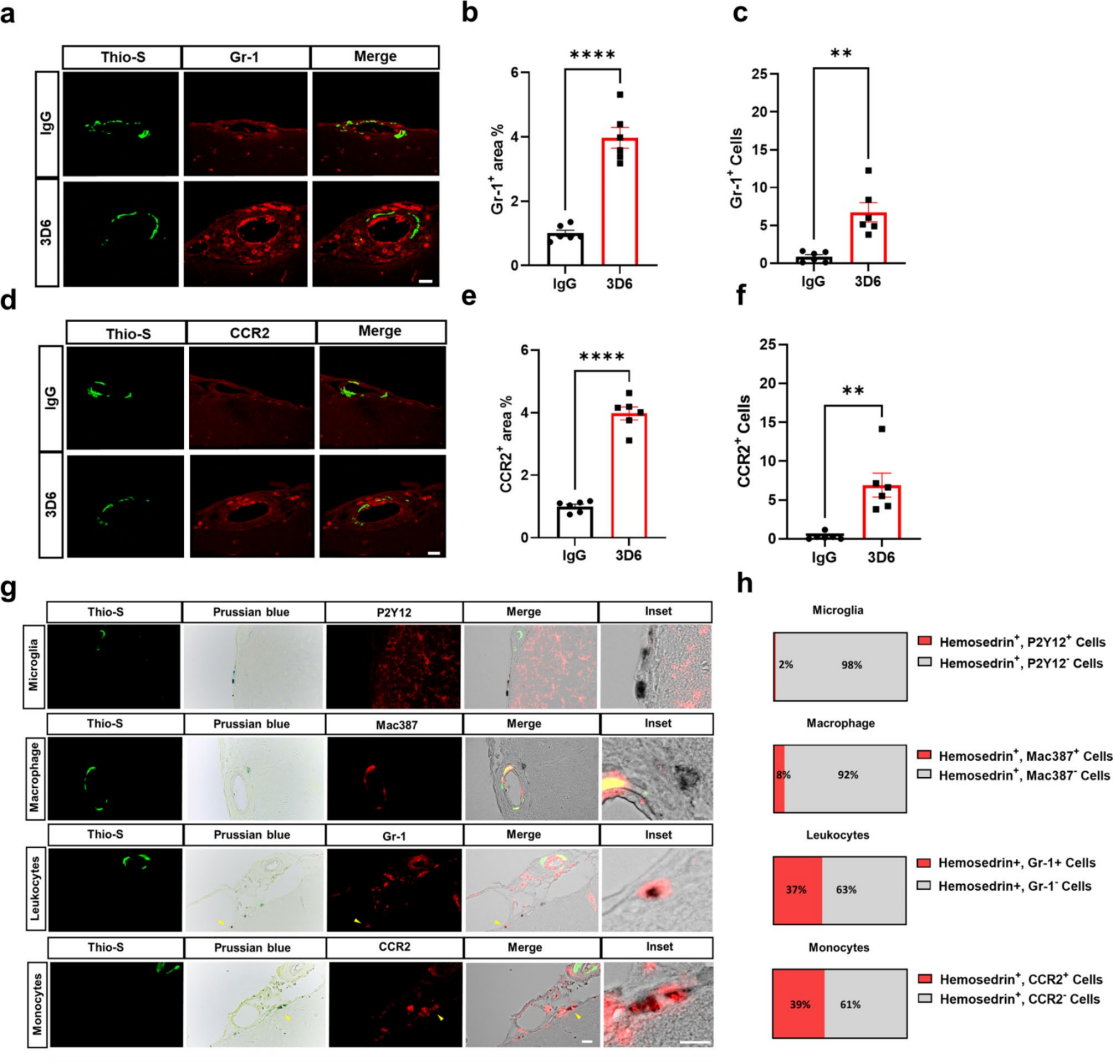
Inflammatory monocytes are highly abundant around vascular amyloid deposits and are associated with microhemorrhages in 3D6 treated PDAPP mice. **(a)** Double immunofluorescence of amyloid (Thio-S, green) and leukocytes (GR-1, red) in PDAPP mice treated with 3D6 or IgG control. Thio-S and GR-1 (red) immunoreactivity overlay (Merge). **(b)** Quantification of GR-1^+^ area (%) of IgG or 3D6 treated mice. **(c)** Quantification of GR-1^+^ cells around vascular amyloid deposits of IgG or 3D6 treated mice. **(d)** Double immunofluorescence of amyloid (Thio-S, green) and monocytes (CCR2, red) in PDAPP mice treated with 3D6 or IgG control. Thio-S and CCR2 (red) immunoreactivity overlay (Merge). **(e)** Quantification of CCR2^+^ area (%) of IgG or 3D6 treated mice. **(f)** Quantification of CCR2^+^ cells around vascular amyloid deposits of IgG or 3D6 treated mice The number of vascular amyloid deposits analyzed was 8–10 per animal. **(g)** Triple labeling of amyloid (Thio-S, green), Prussian Blue (hemosiderin, blue), and immune cells (microglia, macrophages, and monocytes, red) revealed hemosiderin-laden monocytes near CAA-associated microhemorrhages after 3D6 treatment. **(h)** Quantification of the proportion of Hemosiderin^+^ immune cells in 3D6 treated PDAPP mice. For quantifications, eight to seventeen images were used for each experiment n = 6 (mice), and 140–200 cells were counted. Asterisks indicate significant differences, where ** p < 0.01 and ****p < 0.0001 by unpaired Student’s t test. All are representative images of 26-month-old PDAPP mice. Scale bar 10 μm inset or 20 μm merge, respectively

To further characterize the immune cell microenvironment surrounding vascular amyloid deposits after Aβ immunotherapy, we used database for annotation, visualization, and integrated discovery (DAVID) to identify the most significantly upregulated gene ontology (GO) term pathways in BMDM activated by antibody mediated Fc receptor signaling. GO enrichment analysis revealed that BMDM activated through Fc receptor signaling have a GO biological process highly enriched in genes corresponding to inflammatory responses and cell chemotaxis of leukocytes including neutrophils, eosinophils, lymphocytes, and monocytes (Supplemental Fig. 11a, Supplementary Data 3). It is well known that proinflammatory monocytes, characterized by high expression of Ly-6 C (Gr-1) and CC chemokine receptor 2 (CCR2), are recruited to sites of inflammation under various conditions and are predominantly dependent on CCR2 [44, 45]. Importantly, GO molecular functions corresponding to CCR chemokine receptor binding were the most highly enriched overall (Supplemental Fig. 11b, Supplementary Data 3). To determine the presence of infiltrating immune cells, we performed triple staining with Thio-S, PECAM-1, and GR-1 to determine leukocytes association with vascular amyloid accumulation in the leptomeninges. We observed that leukocytes are highly abundant around vascular amyloid in 3D6-treated PDAPP mice with significant increases in GR-1^+^ area and GR-1^+^ cells relative to control IgG (Fig. 5, a-c). To further confirm the leukocytes as monocytes, we performed triple staining with Thio-S, PECAM-1, and CCR2 a specific marker for regulating monocyte infiltration into the brain [46]. Our results confirm monocytes are highly abundant around vascular amyloid in 3D6-treated PDAPP mice, with significant increases in CCR2^+^ area and CCR2^+^ cells relative to control IgG (Fig. 5, d-f). Recent evidence suggests a dual role of monocytes following intracerebral hemorrhage, initially contributing to inflammation leading to secondary damage and, on the other hand, contributing to repair in the clearance of vascular bleeds in perivascular spaces, promoting recovery [47–49]. To determine the association of immune cells with microhemorrhages, we performed triple staining with Thio-S, Prussian blue dye, which identify hemosiderin deposits indicative of previous hemorrhages and microglia, macrophages, and monocytes, respectively (Fig. 5g). Our results show 2% of hemosiderin deposits were immunoreactive for P2Y12^+^ staining and 8% of hemosiderin deposits were immunoreactive for Mac387^+^ staining. Remarkably, we observed infiltrating monocytes were highly associated with hemosiderin deposits in PDAPP mice, with 37% of hemosiderin deposits immunoreactive for Gr-1^+^ staining and 39% of hemosiderin deposits immunoreactive for CCR2^+^ staining (Fig. 5h). These observations indicate 3D6-Aβ immunotherapy induces microhemorrhages in PDAPP mice with an increased abundance of infiltrating monocytes surrounding vascular amyloid deposits and associated with hemosiderin deposits from vascular bleeds.

## Discussion

In the present study, we showed perivascular macrophages can be activated by anti-Aβ antibodies enriched at vascular amyloid deposits. Specifically, 3D6-Aβ immunotherapy promotes the formation of immune complexes with vascular amyloid deposits. These complexes subsequently activate CD169^+^ perivascular macrophages and are associated with increased plasma protein extravasation and microhemorrhages in PDAPP mice. Furthermore, we showed that cultured BMDM activated by antibody-mediated Fc receptor signaling are enriched in inflammatory and extracellular matrix remodeling genes and confirmed the in vivo presence of MMP9 and Timp1-positive perivascular macrophages Finally, we show that hemosiderin deposits of cerebral microhemorrhages are surrounded by inflammatory monocytes, which are remarkably common around vascular amyloid deposits. These findings highlight the crucial involvement of perivascular macrophages in CAA mediated vascular permeability and microhemorrhages associated with amyloid immunotherapy likely mediated through the amplification of the local inflammatory environment and disrupted remodeling of the extracellular milieu surrounding vascular amyloid deposits.

In recent years, disease-modifying treatments for AD have primarily focused on the passive immunization of anti-Aβ antibodies to facilitate the clearance of plaques through the formation of antibody immune complexes which subsequently target amyloid for opsonization and phagocytosis. Bapineuzumab, aducanumab, donanemab and lecanemab were tested in late stage clinical trials with various degrees of efficacy for plaque removal and rescue of cognition decline. Bapineuzumab, a humanized form of murine monoclonal antibody 3D6, was the first antibody used for passive immunotherapy in late-stage clinical trials for AD, recognizing the linear N-terminus of Aβ and binding all forms of Aβ including prefibrillar aggregates and plaques [3]. Although effective in removing plaques, these antibodies have all resulted in ARIA, a serious risk and unintended adverse event manifesting in the human brain as edema (ARIA-E) or microhemorrhages (ARIA-H). Multiple theories have been postulated for the pathogenesis of treatment-related ARIA, as microhemorrhages have been shown to appear regardless of whether immunotherapy is active [19] or passive [20–23]. Nevertheless, accumulating evidence strongly suggests that the presence of vascular amyloid deposition, a primary component of CAA, is a critical factor for the development of ARIA [23]. In CAA, Aβ is deposited within vessel walls along perivascular drainage pathways in the brain, particularly between the endothelial and pial basement membranes (BMs), triggering profound changes in the perivascular space, including altered BM morphology, composition, thickness, and degeneration of smooth muscle cells noted in multiple studies of AD and CAA [50–52]. Data from human patients and in vivo models for CAA suggests that damage to vessel walls activates the endothelium, increases BBB permeability, facilitates the infiltration of monocytes/macrophages, and incites glial reactivity, increasing the production of pro-inflammatory cytokines [53, 54]. Recently, studies have suggested a multifaceted role for perivascular macrophages in the regulation of CAA, as the depletion of perivascular macrophages by directly injecting clodronate-containing liposomes in AD/CAA transgenic mouse models, results in a significant increase in CAA in cortical and leptomeningeal blood vessels [13, 55]. Whereas, the stimulation of perivascular macrophages by injecting chitin, a long-chain polymer of N-Acetylglucosamine, caused a significant reduction of Thio-S labeled cortical blood vessels and CAA load [13]. On the other hand, the sustained activation of perivascular macrophages has also been shown to accelerate neurovascular dysfunction as amyloid-mediated activation stimulates the release of reactive oxygen species, leading to increased cerebrovascular oxidative stress, ultimately suggesting perivascular macrophages as key immunoregulatory cells mediating vascular damage [17]. The utilization of single-cell analysis and fate-mapping techniques in transgenic animal models has greatly aided in the identification of distinct subsets of border-associated macrophages (BAMs). These subsets include subdural/leptomeningeal macrophages, dural macrophages, stromal choroid plexus macrophages, choroid epiplexus macrophages, and perivascular macrophages, each exhibiting unique characteristics in terms of morphology, motility, and function. Despite variations in transcriptional profiles and dynamics among BAMs, a comprehensive understanding of the functional diversity within these subsets in specific anatomical locations remains to be fully understood [56]. Notably, CD169^+^ perivascular macrophages are a unique subset of macrophages present in multiple tissues and organs throughout the body, primarily expressed in secondary lymphoid organs where blood and lymph enter and leave, ideally positioned to detect and respond to potentially harmful foreign agents and serve important roles in phagocytosis, antigen presentation, immune tolerance, and inflammatory responses [32]. These findings are supported by the results of our study, in which we report for the first time that CD169^+^ perivascular macrophages of the CNS are highly associated with vascular amyloid deposits following Aβ immunotherapy, forming an immune complex between basement membranes of leptomeningeal and penetrating vessels of the cortex and play important roles modulating local immune responses. To investigate the broader mechanisms of immune activation in macrophages, we utilized bone marrow-derived macrophages as a model to inference the transcriptional changes occurring in perivascular macrophages activated by Aβ immune complexes. We demonstrated that extracellular matrix remodeling genes were strongly enriched in BMDM activated through Fc receptor signaling, with the most significantly expressed genes corresponding to Timp1 and MMPs. Increased MMP/TIMP protein levels are frequently analyzed and interpreted in respect to the pathophysiology of disease and the net rise in MMP proteolytic activity. Although this interpretation is certainly valid and supports many aspects of disease pathology, such a perspective does not take into account the potential implications of TIMP activity as relevant for mechanisms of tissue injury or repair [57]. In AD patients, an interruption in the balance of MMPs and TIMPs has been studied in detail, as MMP9 is heavily implicated in the occurrence of cerebral hemorrhage due to immunotherapy [40]. Moreover, the MMP-9/Timp-1 ratio is independently associated with cerebral edema [58] and symptomatic intracerebral hemorrhage in ischemic stroke patients [59], indicating a critical role for the MMP9/Timp1 ratio in the regulation of BBB integrity. These findings are supported by the results of our study, in which we confirm the upregulation of MMP9 and Timp1 in perivascular macrophages associated with vascular amyloid deposits, strongly suggesting engagement of perivascular macrophages with vascular amyloid and anti-Aβ antibodies are associated with preponderant extracellular matrix remodeling and leakage of blood plasma proteins. These findings align with the recently clarified role of parenchymal border macrophages in regulating extracellular matrix remodeling in aging and neurodegeneration [60] however, further studies will be necessary to determine the extent to which perivascular macrophages activated by Aβ immunotherapy enhance the MMP9/Timp1 ratio in influencing ARIA.

In addition, gene ontology analysis also revealed inflammatory and chemotactic signaling as the top biological processes and molecular functions of BMDM activated through Fc receptor signaling. To this extent, we observed that Aβ immunotherapy is associated with Ly-6 C^+^ (Gr-1) and CC chemokine receptor 2 (CCR2^+^) proinflammatory monocytes around sites of vascular amyloid deposits. Recent evidence suggests a dual role of monocytes following intracerebral hemorrhage, initially worsening early neurological injury but also contributing to repair in the clearance of vascular bleeds in perivascular spaces, promoting recovery [47–49]. Furthermore, monocyte infiltration into the CNS is a multistep process involving initial penetration of the endothelial monolayer and underlying BM, followed by temporary residency in the perivascular cuff bordered by the endothelial and parenchymal BMs, and finally migration across the parenchymal BM and glia limitans into the brain parenchyma [61]. To this extent, we observed monocytes are highly associated with hemosiderin deposits in the brain parenchyma of PDAPP mice following Aβ immunotherapy with 37% of hemosiderin deposits colocalizing with Gr-1^+^ cells and 39% of hemosiderin deposits colocalizing with CCR2^+^ cells. This finding expands on the traditional notion that macrophages are solely implicated in hemosiderin deposits. Interestingly, production of MMPs by perivascular macrophages has been demonstrated to promote monocyte infiltration into the brain parenchyma as macrophage-derived MMP2 and MMP9 activity is crucial for leukocyte penetration of the parenchymal BM [61]. These observations of the temporal impact of perivascular macrophage activity on cerebrovascular dysfunction supports our findings of transitory plasma protein extravasation of fibrinogen and long-term observations of monocyte recruitment to cerebral microbleeds. Therefore, considering our primary culture-based results that show BMDM activated by Fc receptor signaling are enriched in extracellular matrix remodeling genes and Aβ immunotherapy is associated with MMP9/Timp1-positive perivascular macrophages associated with antibody/amyloid complexes, it is reasonable to suggest that engagement of perivascular macrophages with vascular amyloid is sufficient to elicit extracellular matrix remodeling in the perivascular compartment, implicating perivascular macrophages in tissue remodeling and vascular dysfunction associated with altered vascular permeability and microhemorrhages during Aβ immunotherapy.

Despite the remaining uncertainties surrounding the precise mechanism underlying the association between perivascular macrophage activation and ARIA-E and ARIA-H our understanding of this relationship is further complicated by the limited availability of mouse models for studying ARIA-E. Nevertheless, based on transgenic mouse studies, two plausible hypotheses have been proposed to explain the clinical manifestation of ARIA-H, suggesting that microhemorrhage could be attributed to either the redistribution of Aβ into cerebral blood vessels [21], or the direct binding of antibodies to existing CAA [20]. The observed reduction in 3D6 immunoreactivity as a result of anti-amyloid immunotherapy provides compelling evidence that the observed changes are indeed linked to immune complex formation, rather than redistribution of Aβ from brain parenchyma to cerebral vasculature. This finding further reinforces the results of our present study, which suggests that the direct binding of antibodies to vascular amyloid and subsequent activation of perivascular macrophages play a pivotal role in enhancing cerebral amyloid angiopathy (CAA)-mediated vascular permeability and microhemorrhages associated with amyloid immunotherapy. It is highly plausible that this effect is mediated through the amplification of the local inflammatory environment and the disruption of extracellular matrix remodeling around vascular amyloid deposits.

## Conclusions

Overall, our findings support the idea that Aβ antibodies enriched at CAA deposits leads to the recruitment and activation of perivascular macrophages, enhancing vascular permeability and susceptibility to microhemorrhages. We confirmed that macrophages activated by antibody mediated Fc receptor signaling are enriched in inflammatory signaling and extracellular matrix remodeling genes and confirmed the in vivo presence of Timp1^+^ CD169^+^ and MMP9^+^ CD169^+^ perivascular macrophages associated with vascular amyloid deposits in mouse models of AD. Finally, we provide evidence to suggest that CD169^+^ perivascular macrophages may enhance CAA-mediated vascular permeability, plasma protein extravasation, and infiltrating monocytes associated with microhemorrhages. Nevertheless, we acknowledge the use of BMDM in our study is a limitation, as BMDM were grown in media containing serum known to impact changes in gene expression and protein levels, the use of as BMDM merely provides a framework to highlight the broader mechanisms of immune activation in macrophages. To delve deeper into these differences, we recognize that conducting further transcriptomic analyses using isolated and enriched perivascular macrophages, as well as human brain samples, especially clinical samples collected from immunotherapy trials, will be imperative. By doing so, we can comprehensively dissect the differences in detail, most importantly, establish the precise role of perivascular macrophages in the human brain during Aβ immunotherapy.

## Methods

### Transgenic mouse model

PDAPP, hTau APP KI, and wild type C57BL/6J (WT) male and female mice were used in our experiments, including cellular and immunohistochemistry (IHC) analyses. PDAPP colony (line 13388) was established through an inbreeding exercise wherein mice were inbred from selected litters that maintained decreased variability in both soluble and insoluble Aβ. The plaque deposition phenotype of the inbred PDAPP line 13388 was similar to the originally described PDAPP colony [20]. The pathology of *APP KI* mice is characterized by predictable plaque development with progressive increases in parenchymal amyloid pathology as well as mild but detectable cerebral amyloid angiopathy [62]. PDAPP mice received weekly subcutaneous injections of 3D6 (anti-Aβ1-x,IgG2b) (25 mg/kg) or twice-weekly subcutaneous injections of IgG (IgG2a isotype) (50 mg/kg) for three months. hTau APP KI mice received weekly subcutaneous injections of biotynlated-3D6 (anti-Aβ1-x,IgG2b) (25 mg/kg) or weekly subcutaneous injections of IgG (IgG2a isotype) (25 mg/kg) for one month. For dosing experiments, 23-26-month-old were utilized. This age was selected due to the extensive vascular amyloid accumulation at 23–26 months of age in the PDAPP and hTau APP KI models. All experiments were performed in accordance with the Institutional Animal Care and Use Guidelines for Eli Lilly.

### Brain sections immunofluorescence

Mice were anesthetized with avertin and perfused with ice-cold PBS. Brains were removed from the cranium and fixed in 4% (wt/vol) paraformaldehyde (PFA) for 24 h followed by cryoprotection in 30% (wt/vol) sucrose in PBS solution. Brains were subsequently frozen and coronally sectioned (10 μm thick) using a freezing/sliding microtome. Paraffin embedded coronal sections (10 μm thick) were deparaffinized in xylene, rehydrated in ethanol (EtOH) and washed with deionized water. Then, sections were heated in low pH 1x citrate buffer for 30 min. After washing in PBS for 5 min twice, the sections were blocked with a solution of 5% goat serum and 0.01% Triton X-100 in PBS for 1 h at room temperature (RT). Sections were then incubated overnight at 4 °C with the following antibodies, each diluted 1:100 in blocking solution: anti-P2Y12 (as-55043a, AnaSpec), anti-Mac387 (MA1-81381, ThermoFisher), anti-Clec7a (internally generated), anti-CD206 (NC9751189, ThermoFisher), anti-CD169 (NB600-534, NovusBio), anti-CD169 (MA1-80164), anti-CD169 (PIPA5115898), anti-CD163 (50-198-4796, ThermoFisher) anti-Laminin (NB300-144,NovusBio) anti-CD 31 (PA5-16301, ThermoFisher), anti-CD 31 (14-0311-82, ThermoFisher), anti-Timp1 (16644-1-ap, Proteintech), anti-GFAP (13-030-0), anti-MMP9 (ab38898, Abcam), anti-Fibrinogen (ab34269, Abcam), anti-GR-1 (14-5931-82, ThermoFisher), and anti-CCR2 (MA5-41175, ThermoFisher). The next day, the sections were washed 3 times in PBS and incubated with the following secondary antibodies, 1 h at room temperature, diluted 1:100 in blocking solution: goat anti-mouse IgG 555 (A31570, ThermoFisher), goat anti-mouse IgG 647 (A32728, ThermoFisher), goat anti-rabbit IgG 555 (A32732, ThermoFisher), goat anti-rabbit IgG 647 (A32733, ThermoFisher), goat anti-rat IgG 555 (A21434, ThermoFisher), goat anti-rat IgG 647 (A48272, ThermoFisher), streptavidin 488 (S32354, ThermoFisher). Slides were then crosslinked with 4% pfa for 5 min followed by two 5-min washes in deionized PBS. Amyloid was stained with 0.025% Thioflavin-S (Thio-S, Sigma-Aldrich, T1892) or X-34 (SML1954, 1:10,000) prepared in 50% EtOH in TBS for 10 min at RT, followed by two 5-min washes in 50% EtOH and two 5-min washes in PBS. Finally, the sections were washed in PBS and mounted with ProLong Diamond Antifade medium without DAPI (P36961, ThermoFisher).

### Immunohistochemistry and chromogenic staining

Slides were stained for vascular bleeds marked by hemosiderin deposits by incubating in 2% potassium ferrocyanide (EMS, 26613-01) in 2% hydrochloric acid for 30 min followed by two 5-min washes in PBS. Slides utilized for microhemorrhage analysis were counter stained with nuclear fast-red (N8002, Sigma-Aldrich) for 5 min followed by a 5-min wash in PBS. Amyloid was stained with 3 µg/ml of biotinylated 3D6. Secondary HRP reagents specific for biotin were employed and the deposited plaque was visualized with DAB-Plus (DAKO). Finally, the sections were washed in PBS and mounted with ProLong Diamond Antifade medium without DAPI (P36961, ThermoFisher).

### Bone marrow derived macrophage primary cell culture

The procedure used for BMDM cultures was based on a previously described approach [63]. Briefly, femurs and tibias were extracted from 8-month-old C57BL/6J mice, submerged in ice cold RPMI media (22400-071), then soaked in 70% EtOH for 10 s and immediately washed with RPMI. To expel the bone marrow, bone epiphyses were cut open and flushed into a conical tube with 5mL of DPBS using a syringe and 26-gauge needle. Bone marrow was then collected and passed through a 70 μm cell strainer and resuspended in 37 C prewarmed BMDM media (RPMI, 10% dialyzed FBS, 1x Pen/Strep, 1 mM sodium pyruvate, 1x MEM NEAA, 1x Glutamax). Bone marrow cells were counted and seeded in 10 cm petri dishes at 4E6 cells/plate in 8mL of BMDM media + 50ng/mL of murine macrophage colony stimulating factor (M-CSF, Peprotech, 315-02-50UG).

### Bone marrow derived macrophage antibody engagement

Antibody coated plates were prepared using 24 well plates coated with 200 uL of 10ug/mL IgG2a, mIgG2a.LALAPG or left untreated (PBS vehicle) and sealed with parafilm and incubated overnight at 4 C. After 5 days of culture (37 C, 5% CO2) differentiated BMDMs were washed with PBS and detached with 5mL of 5mM EDTA for 5 min at 37 C. BMDMs were then combined and centrifuged at RT for 5 min at 1500 rpm. Cells were washed with PBS and spun at RT for 5 min at 1500 rpm. The pellet was resuspended in BMDM media and counted. BMDMs were seeded at 400,000 cells/well in the coated 24 well plate in 1mL BMDM media + 20ng/mL murine M-CSF. After 48 h RNA was isolated with a Qiagen Rneasy Plus Micro Kit (74034).

### NanoString gene expression analysis

Total mRNA was purified from primary bone marrow-derived macrophages and multiplexed using the nCounter analysis system (NanoString Technologies, Seattle, WA, USA) combined with the nCounter Mouse Neuroinflammation Panel that includes 770 genes covering the core pathways that define neuroimmune interactions and activation. Briefly, 100 ng total RNA per sample (20 ng/µl) (*n* = 1) was loaded and hybridized with probes for 16 h at 65° C following the manufacturer’s protocol. Counts for target genes were normalized to the best fitting housekeeping genes as determined by nSolver software to account for variation in RNA content. The background signal was calculated as the mean value of the negative hybridization control probes. The expression data were excluded when they had lower than average background signals from the negative controls, and probes with < 100 reads for 6 or more samples were removed from the analysis. The Benjamini-Yekutelli method was used to calculate the false discovery rate (FDR) [64] Significant genes for undirected differential expression were identified using adjusted p-value < 0.01. Downstream analyses and visualizations of gene expression datasets were performed using the ROSALIND analysis platform (OnRamp Bioinformatics) software. GO and KEGG enrichment analyses were performed using the Database for Annotation, Visualization, and Integrated Discovery (DAVID) website [65, 66].

### qPCR

Total RNA was isolated from treated BMDM cultures (50,000 cells using three biological/three technical replicates) with the RNeasy Plus Micro Kit (Qiagen, 74034). cDNA was prepared from 50ng total RNA with Fluidigm Reverse Transcription Master Mix (Standard BioTools, 100–6298). cDNA was pre-amplified with TaqMan PreAmp Master Mix (Applied Biosystems, 4391128) using manufacturer recommended protocol. All qPCRs were performed with a 48.48 Dynamic Array IFC for Gene Expression (Standard BioTools, BMK-M-48.48) on the Biomark HD system. The BMDM relative gene expression was evaluated with the delta Ct method using Taqman probe sets (Actb: Mm02619580_g1, Msr1: Mm00446214_m1, Pdpn: Mm01348912_g1, Timp1: Mm01341361_m1; Applied Biosystems) and TaqMan Universal PCR Master Mix (Applied Biosystems, 4326708).

### Microscopy and image analysis

For image analysis of mouse brain sections, we used ImageJ software v1.53 (NIH) to create one index that represented changes in the number of P2Y12^+^, Mac387 ^+^, CD169 ^+^, PECAM-1 ^+^, Timp1 ^+^, MMP9 ^+^, Fibrinogen ^+^, GR-1 ^+^ or CCR2 ^+^ pixels divided by the total number of pixels in the image, expressed as (+) area %. To ensure the representativeness of our study’s findings, we applied random selection when choosing animals from the total population for subsequent immunohistological investigations. A subgroup of 3D6 and IgG treated PDAPP mice (6 animals each) was randomly selected across microhemorrhage groups and cerebral cortices were examined using a SP8 confocal microscope (Leica), 63x objective with a 0.15 μm z-step. We analyzed 8–10 vascular amyloid deposits from both the leptomeninges and penetrating vessels per animal, using two brain sections per mouse. Furthermore, we quantified the leptomeningeal diameters in microns by measuring from the border of the tissue edge to the pial surface. Results are shown as ± SEM of n = 6 (mice). For colocalization quantification, images were analyzed in Imaris v8.4 (Bitplane) to determine relative colocalization coefficients (colocalization ratio) using Manders’ coefficients [67]. Whole brain slices were scanned using the Zeiss AxioScan Z.1 with a 20X objective (0.8NA). Images were then analyzed for region of interest defined along images of tissue edges (100 μm wide) and Hemosiderin^+^ signal was thresholded to quantify percent coverage of stain relative to ROI using HALO v3.5 image analysis suite (Indica Labs). Brightfield images were acquired using Olympus BX63 with a 40X objective and cells were counted using ImageJ v1.53.

### Statistics and reproducibility

The details about experimental design and statistics used in different data analyses performed in this study are given in the respective sections of results and methods. Sample sizes were determined based on previous publications. NanoString experiments were conducted with four technical replicates, and qPCR was validated with three biological replicates. For imaging experimental modalities utilized in this study, a minimum of six independent biological replicates were employed to ensure robust and reliable results. Investigators were blinded for staining experiments. GraphPad Prism 8.0.2 was used to perform all statistical analyses. Statistical significance between groups was calculated using a student’s *t*-test (two-tailed). Data is presented as the mean ± SEM unless otherwise stated. *, **, *** and **** denote p < 0.05, p < 0.01, p < 0.001 and p < < 0.0001, respectively. No other statistical comparisons were significant unless otherwise noted.

### Electronic Supplementary Material

Below is the link to the electronic supplementary material.

Supplemental Fig. 1: Reduced 3D6 immunoreactivity in anti-amyloid immunotherapy treated PDAPP mice. (a) Parenchymal and vascular amyloid Aβ deposits (brown, 3D6) across coronal sections of PDAPP mice treated with IgG or 3D6. (b) Quantification of 3D6^+^ area (%) in brain coronal sections of IgG or 3D6-treated mice. (c) Parenchymal Aβ deposits quantification of 3D6^+^ area (%) in brain coronal sections of IgG or 3D6-treated mice. (d) Vascular Aβ deposits quantification of 3D6^+^ area (%) in brain coronal sections of IgG or 3D6-treated mice. Each data point indicates an animal n = 20–25 (mice). All are representative images of 26-month-old PDAPP mice. Results are shown as ± SEM, asterisks indicate significant differences, where **p < 0.01 and *** p < 0.001 by unpaired Student’s t test. Scale bar 20 or 500 μm, respectively.

Supplemental Fig. 2: Perivascular macrophages are highly associated with vascular amyloid of penetrating vessels 3D6 treated PDAPP Mice. (a) Triple immunofluorescence of amyloid (Thio-S, green), microglia (P2Y12, red) and endothelial cells (PECAM-1, cyan) in PDAPP mice treated with 3D6 or IgG control. Thio-S, P2Y12 and PECAM-1 immunoreactivity overlay (Merge). Colocalization analysis (white). (b) Quantification of P2Y12^+^ area (%) of IgG or 3D6 treated mice. (c) Quantification of colocalization ratio of Thio-S and P2Y12. (d) Triple immunofluorescence of amyloid (Thio-S, green), macrophages (Mac387, red) and endothelial cells (PECAM-1, cyan) in PDAPP mice treated with 3D6 or IgG control. Thio-S, Mac387 and PECAM-1 immunoreactivity overlay (Merge). Colocalization analysis (white). (e) Quantification of Mac387^+^ area (%) of IgG or 3D6 treated mice. (f) Quantification of colocalization ratio of Thio-S and Mac387. (g) Triple immunofluorescence of amyloid (Thio-S, green), perivascular macrophages (CD169, red) and endothelial cells (PECAM-1, cyan) in PDAPP mice treated with 3D6 or IgG control. Thio-S, CD169 and PECAM-1 immunoreactivity overlay (Merge). Colocalization analysis (white). (h) Quantification of CD169^+^ area (%) of IgG or 3D6 treated mice. (i) Quantification of colocalization ratio of Thio-S and CD169. The number of vascular amyloid deposits analyzed was 8–10 per animal. Results are shown as ± SEM of n = 6 (mice). Asterisks indicate significant differences, where **p < 0.01 and ****p < 0.0001 by unpaired Student’s t test. Scale bar 5 μm CC or 10 μm merge, respectively.

Supplemental Fig. 3: Clec7a^+^ microglia are not associated with vascular amyloid. (a) Double immunofluorescence of amyloid (Thio-S, green), and activated microglia (Clec7a, red) in leptomeninges of PDAPP mice treated with 3D6 or IgG control. Thio-S and Clec7a immunoreactivity overlay (Merge). (b) Double immunofluorescence of amyloid (Thio-S, green), and activated microglia (Clec7a, red) in penetrating vessels of PDAPP mice treated with 3D6 or IgG control. Thio-S and Clec7a immunoreactivity overlay (Merge). Scale bar 10 μm.

Supplemental Fig. 4: Perivascular macrophages exhibit enrichment exclusively with amyloid^+^ vessels in 3D6 treated PDAPP Mice. (a) Triple immunofluorescence of amyloid^+^ (Thio-S, green) or amyloid^−^ vessels, perivascular macrophage (CD169, red) and endothelial cells (PECAM-1, cyan) in PDAPP mice treated with IgG control. Thio-S, CD169 and PECAM-1 immunoreactivity overlay (Merge). (b) Quantification of CD169^+^ area (%) of IgG in amyloid^+^ or amyloid^−^ vessels. (c) Triple immunofluorescence of amyloid^+^ (Thio-S, green) or amyloid^−^ vessels, perivascular macrophage (CD169, red) and endothelial cells (PECAM-1, cyan) in PDAPP mice treated with 3D6. Thio-S, CD169 and PECAM-1 immunoreactivity overlay (Merge). (d) Quantification of CD169^+^ area (%) of 3D6 in amyloid^+^ or amyloid^−^ vessels. The number of vessels analyzed was 8–10 per animal. Results are shown as ± SEM of n = 6 (mice). Asterisks indicate significant differences, where ***p < 0.001 by unpaired Student’s t test. Scale bar 10 μm merge or 5 μm inset, respectively.

Supplemental Fig. 5 Perivascular macrophages associated with vascular amyloid are CD206 and CD163 positive. Triple immunofluorescence of amyloid (X-34, blue), perivascular macrophages (CD206, red) (CD163, green) in PDAPP mice treated with 3D6 or IgG control. X-34, CD206 and CD163 immunoreactivity overlay (Merge). Scale bar 5 μm.

Supplemental Fig. 6 qPCR confirms dysregulated inflammatory signaling and extracellular matrix remodeling genes. (a) Relative expression of Pdpn in BMDM activated by IgG2a vs. control LALAPG (b) Relative expression of Timp1 in BMDM activated by IgG2a vs. control LALAPG (c) Relative expression of Msr1 in BMDM activated by IgG2a vs. control LALAPG. Data was normalized to the levels of actin mRNA. Relative quantitation was performed using 2^−ΔΔCt^ (fold change) method. Results are shown as the mean ± SEM of n = 3 (independent cultures). Asterisks indicate significant differences, where * p < 0.05 and ** p < 0.01.

Supplemental Fig. 7 Bone marrow-derived macrophages plated with LALAPG have no significant differential gene expression from non-treatment group. (a) Schematic diagram showing macrophages treated with immunodeficient LALAPG control antibodies. (b) Volcano plots showing not significantly differentially expressed genes in LALAPG activated macrophages vs. control nontreated macrophages. Horizontal line values correspond to p-value < 0.01(solid), p-value < 0.05 (dashed).

Supplemental Fig. 8 Aβ immunotherapy activated perivascular macrophages of penetrating vessels express extracellular matrix remodeling genes. (a) Triple immunofluorescence of amyloid (Thio-S, green), Timp1 (red) and perivascular macrophages (CD169, cyan) in PDAPP mice treated with 3D6 or IgG control. Thio-S, Timp1 and CD169 immunoreactivity overlay (Merge). Colocalization analysis (white). (b) Quantification of Timp1^+^ area (%) of IgG or 3D6 treated mice. (c) Quantification of colocalization ratio of Timp1 and CD169. (d) Triple immunofluorescence of amyloid (Thio-S, green), MMP9 (red) and perivascular macrophages (CD169, cyan) in PDAPP mice treated with 3D6 or IgG control. Thio-S, MMP9 and CD169 immunoreactivity overlay (Merge). Colocalization analysis (white). (e) Quantification of MMP9^+^ area (%) of IgG or 3D6 treated mice. (f) Quantification of colocalization ratio of MMP9 and CD169. The number of vascular amyloid deposits analyzed was 8–10 per animal. Results are shown as ± SEM of n = 6 (mice). Asterisks indicate significant differences, where *p < 0.05 and ***p < 0.001 by unpaired Student’s t test. Scale bar 5 μm CC or 10 μm merge, respectively.

Supplemental Fig. 9 Timp1^+^ immunoreactivity was not detected in astrocytes, microglia, or endothelial cells. (a) Four color immunofluorescence of amyloid (X-34, blue), astrocytes (GFAP, green), Timp1( red) and endothelial cells (PECAM-1) in PDAPP mice treated with 3D6 or IgG control. (b) Four color immunofluorescence of amyloid (X-34, blue), microglia (Clec7a, green), Timp1 (red) and endothelial cells (PECAM-1) in PDAPP mice treated with 3D6 or IgG control. Immunoreactivity overlay (Merge). Scale bar 10 μm.

Supplemental Fig. 10 Increased fibrinogen around vascular amyloid deposits in 3D6 treated PDAPP Mice. (a) Triple immunofluorescence of amyloid (Thio-S, green), fibrinogen (red) and endothelial cells (PECAM-1, cyan) in the leptomeninges of PDAPP mice treated with 3D6 or IgG control. Thio-S, fibrinogen, and endothelial cells (PECAM-1, cyan) immunoreactivity overlay (Merge). (b) Quantification of fibrinogen^+^ area (%) of IgG or 3D6 treated mice. (c) Triple immunofluorescence of amyloid (Thio-S, green), fibrinogen (red) and endothelial cells (PECAM-1, cyan) of penetrating vessels PDAPP mice treated with 3D6 or IgG control. Thio-S, fibrinogen, and PECAM-1 immunoreactivity overlay (Merge). (d) Quantification of fibrinogen^+^ area (%) of IgG or 3D6 treated mice. The number of vascular amyloid deposits analyzed was 8–10 per animal. Results are shown as ± SEM of n = 6 (mice). Asterisks indicate significant differences, where ** p < 0.01 and ***p < 0.001 by unpaired Student’s t test. Scale bar 5 μm merge or 10 μm inset, respectively.

Supplemental Fig. 11 Macrophages activated by Fc receptor signaling have biological processes and molecular functions enriched in chemotactic signaling. (a,b) DAVID bioinformatics gene ontology enrichment analysis of differentially expressed genes upregulated in bone marrow-derived macrophages activated by Fc receptor signaling.

## Data Availability

The NanoString data supporting the conclusions of this article are available at NCBI’s Gene Expression Omnibus (GEO) and is accessible via series accession numbers GSE222466.

## Abbreviations

Aβ: Amyloid-β
AD: Alzheimer disease
APP: Amyloid precursor protein
ARIA: Amyloid-related imaging abnormalities
ARIA-E: (Amyloid-Related Imaging Abnormalities with Edema)
ARIA-H: (Amyloid-Related Imaging Abnormalities with Hemorrhage)
BAM: Border-associated macrophages
BBB: Blood brain barrier
BM: Basement membrane
BMDM: Bone marrow-derived macrophages
CAA: Cerebral amyloid angiopathy
CCL: CC chemokine ligands
CCR: CC chemokine receptor
CNS: Central nervous system
GSEA: Gene set enrichment analysis
IgG: Immunoglobulin G
KI: Knock-in
MMP9: Matrix metallopeptidase 9
TIMP1: Tissue inhibitor matrix metalloproteinase 1
