# Supplementary material for: Amyloid-β (Aβ) immunotherapy induced microhemorrhages are associated with activated perivascular macrophages and peripheral monocyte recruitment in Alzheimer’s disease mice
Source: Mol Neurodegener. 2023 Aug 30;18:59. doi: 10.1186/s13024-023-00649-w (PMC10469415; doi:10.1186/s13024-023-00649-w)
Supplement: Supplementary file 1 — Supplemental Fig. 1: Reduced 3D6 immunoreactivity in anti-amyloid immunotherapy treated PDAPP mice. (a) Parenchymal and vascular amyloid Aβ deposits (brown, 3D6) across coronal sections of PDAPP mice treated with IgG or 3D6. (b) Quantification of 3D6+ area (%) in brain coronal sections of IgG or 3D6-treated mice. (c) Parenchymal Aβ deposits quantification of 3D6+ area (%) in brain coronal sections of IgG or 3D6-treated mice. (d) Vascular Aβ deposits quantification of 3D6+ area (%) in brain coronal sections of IgG or 3D6-treated mice. Each data point indicates an animal n = 20–25 (mice). All are representative images of 26-month-old PDAPP mice. Results are shown as ± SEM, asterisks indicate significant differences, where **p < 0.01 and *** p < 0.001 by unpaired Student’s t test. Scale bar 20 or 500 μm, respectively. [file 13024_2023_649_MOESM1_ESM.docx]

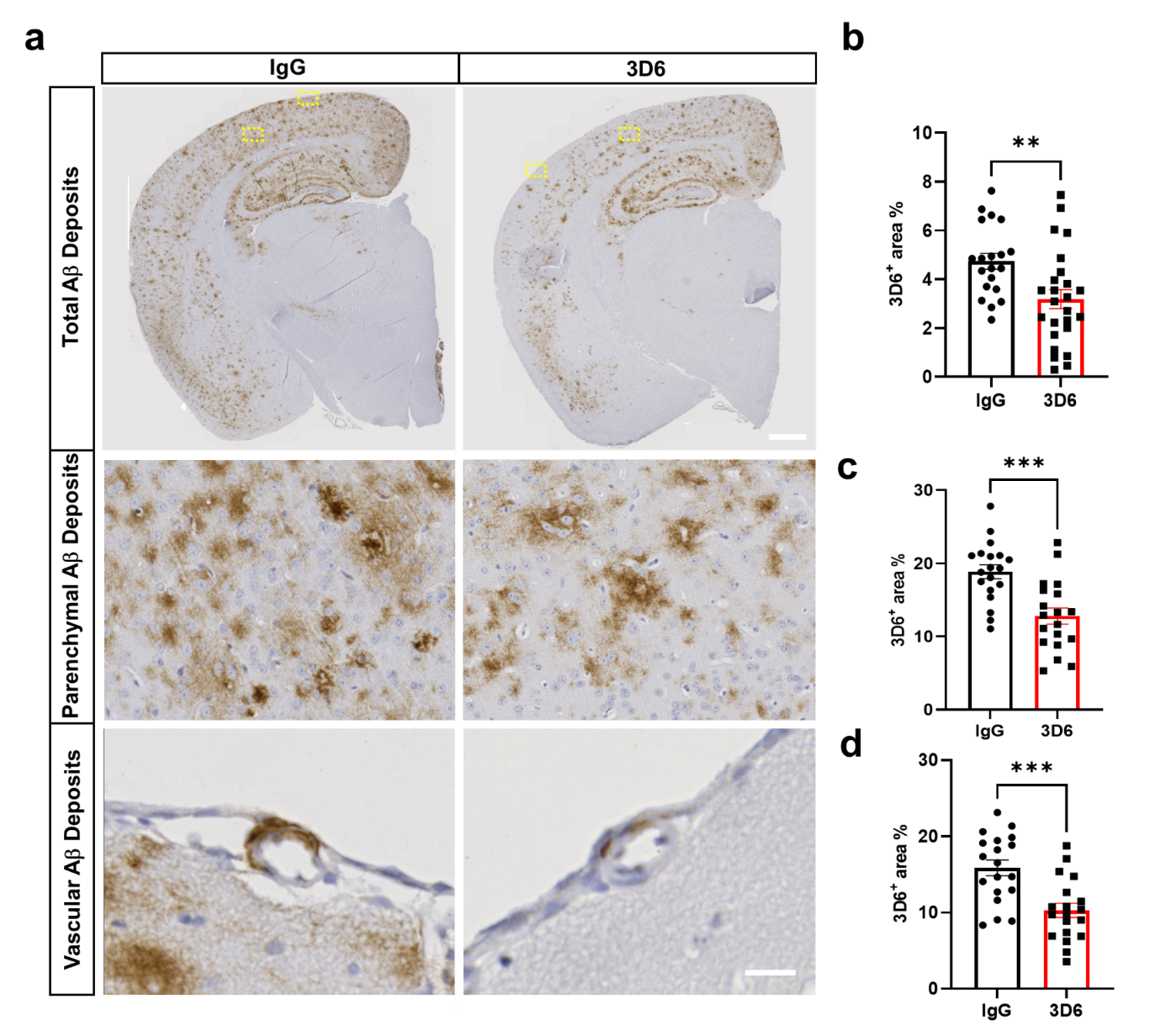
**Supplemental Figure 1. Reduced 3D6 immunoreactivity in anti-amyloid immunotherapy treated PDAPP mice. (a)** Parenchymal and vascular amyloid Aβ deposits (brown, 3D6) across coronal sections of PDAPP mice treated with IgG or 3D6. **(b)** Quantification of 3D6^+^ area (%) in brain coronal sections of IgG or 3D6-treated mice. **(c)** Parenchymal Aβ deposits quantification of 3D6^+^ area (%) in brain coronal sections of IgG or 3D6-treated mice. **(d)** Vascular Aβ deposits quantification of 3D6^+^ area (%) in brain coronal sections of IgG or 3D6-treated mice. Each data point indicates an animal n=20-25 (mice). All are representative images of 26-month-old PDAPP mice. Results are shown as ± SEM, asterisks indicate significant differences, where **p < 0.01 and *** p<0.001 by unpaired Student's t test. Scale bar 20 or 500 μm, respectively.
