# Supplementary material for: Amyloid-β (Aβ) immunotherapy induced microhemorrhages are associated with activated perivascular macrophages and peripheral monocyte recruitment in Alzheimer’s disease mice
Source: Mol Neurodegener. 2023 Aug 30;18:59. doi: 10.1186/s13024-023-00649-w (PMC10469415; doi:10.1186/s13024-023-00649-w)
Supplement: Supplementary file 2 — Supplemental Fig. 2: Perivascular macrophages are highly associated with vascular amyloid of penetrating vessels 3D6 treated PDAPP Mice. (a) Triple immunofluorescence of amyloid (Thio-S, green), microglia (P2Y12, red) and endothelial cells (PECAM-1, cyan) in PDAPP mice treated with 3D6 or IgG control. Thio-S, P2Y12 and PECAM-1 immunoreactivity overlay (Merge). Colocalization analysis (white). (b) Quantification of P2Y12+ area (%) of IgG or 3D6 treated mice. (c) Quantification of colocalization ratio of Thio-S and P2Y12. (d) Triple immunofluorescence of amyloid (Thio-S, green), macrophages (Mac387, red) and endothelial cells (PECAM-1, cyan) in PDAPP mice treated with 3D6 or IgG control. Thio-S, Mac387 and PECAM-1 immunoreactivity overlay (Merge). Colocalization analysis (white). (e) Quantification of Mac387+ area (%) of IgG or 3D6 treated mice. (f) Quantification of colocalization ratio of Thio-S and Mac387. (g) Triple immunofluorescence of amyloid (Thio-S, green), perivascular macrophages (CD169, red) and endothelial cells (PECAM-1, cyan) in PDAPP mice treated with 3D6 or IgG control. Thio-S, CD169 and PECAM-1 immunoreactivity overlay (Merge). Colocalization analysis (white). (h) Quantification of CD169+ area (%) of IgG or 3D6 treated mice. (i) Quantification of colocalization ratio of Thio-S and CD169. The number of vascular amyloid deposits analyzed was 8–10 per animal. Results are shown as ± SEM of n = 6 (mice). Asterisks indicate significant differences, where **p < 0.01 and ****p < 0.0001 by unpaired Student’s t test. Scale bar 5 μm CC or 10 μm merge, respectively. [file 13024_2023_649_MOESM2_ESM.docx]

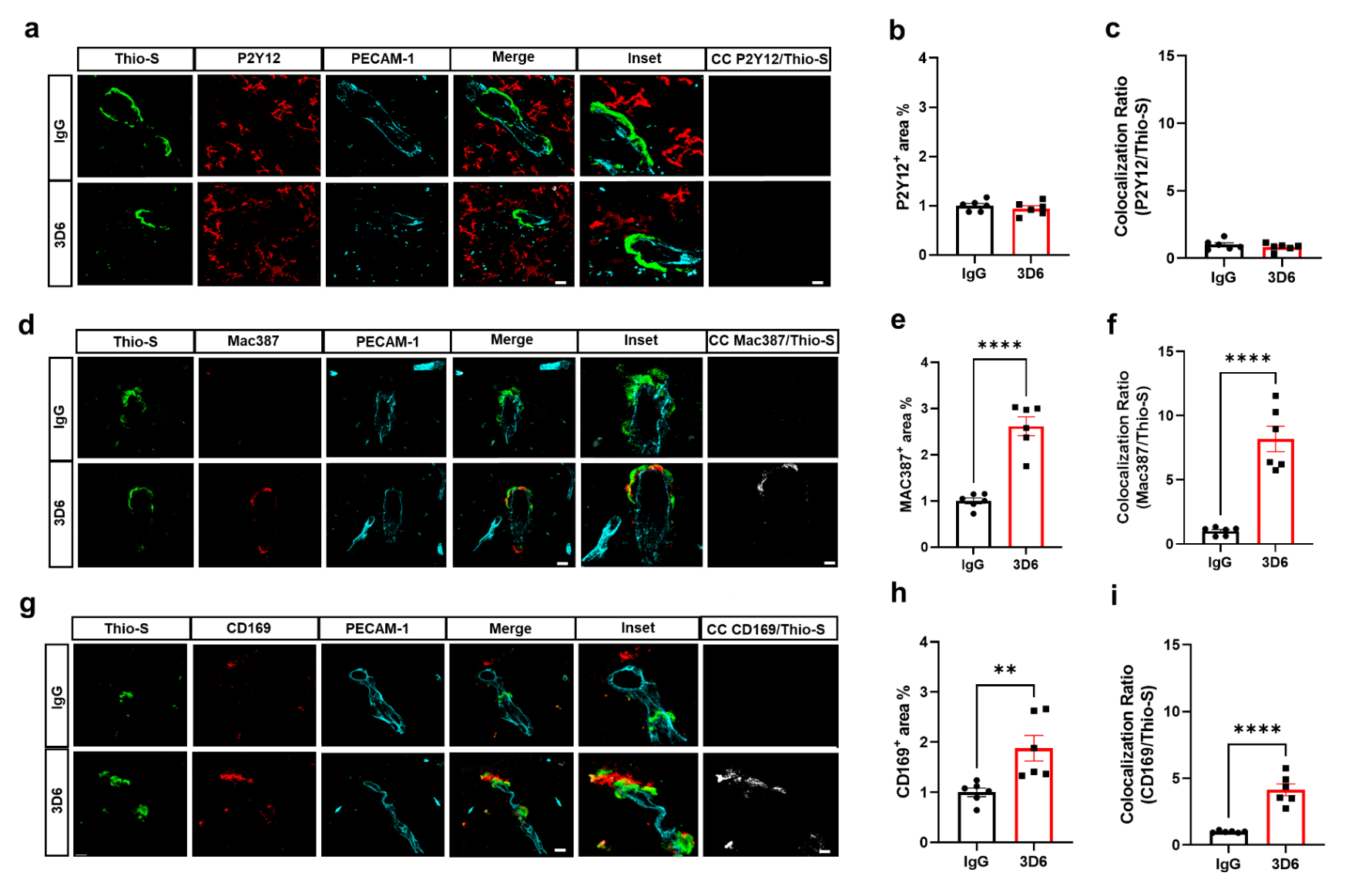


**Supplemental Figure 2. Perivascular macrophages are highly associated with vascular amyloid of penetrating vessels 3D6 treated PDAPP Mice**. (**a)** Triple immunofluorescence of amyloid (Thio-S, green), microglia (P2Y12, red) and endothelial cells (PECAM-1, cyan) in PDAPP mice treated with 3D6 or IgG control. Thio-S, P2Y12 and PECAM-1 immunoreactivity overlay (Merge). Colocalization analysis (white). (**b)** Quantification of P2Y12^+^ area (%) of IgG or 3D6 treated mice. (**c)**Quantification of colocalization ratio of Thio-S and P2Y12. (**d)** Triple immunofluorescence of amyloid (Thio-S, green), macrophages (Mac387, red) and endothelial cells (PECAM-1, cyan) in PDAPP mice treated with 3D6 or IgG control. Thio-S, Mac387 and PECAM-1 immunoreactivity overlay (Merge). Colocalization analysis (white). (**e)** Quantification of Mac387^+^ area (%) of IgG or 3D6 treated mice. (**f)** Quantification of colocalization ratio of Thio-S and Mac387. (**g)** Triple immunofluorescence of amyloid (Thio-S, green), perivascular macrophages (CD169, red) and endothelial cells (PECAM-1, cyan) in PDAPP mice treated with 3D6 or IgG control. Thio-S, CD169 and PECAM-1 immunoreactivity overlay (Merge). Colocalization analysis (white). (**h)** Quantification of CD169^+^ area (%) of IgG or 3D6 treated mice. (**i)** Quantification of colocalization ratio of Thio-S and CD169. The number of vascular amyloid deposits analyzed was 8-10 per animal. Results are shown as ± SEM of n = 6 (mice). Asterisks indicate significant differences, where ***p* < 0.01 and ****p < 0.0001 by unpaired Student's t test. Scale bar 5 μm CC or 10 μm merge, respectively.
