# Supplementary material for: Amyloid-β (Aβ) immunotherapy induced microhemorrhages are associated with activated perivascular macrophages and peripheral monocyte recruitment in Alzheimer’s disease mice
Source: Mol Neurodegener. 2023 Aug 30;18:59. doi: 10.1186/s13024-023-00649-w (PMC10469415; doi:10.1186/s13024-023-00649-w)
Supplement: Supplementary file 3 — Supplemental Fig. 3: Clec7a+ microglia are not associated with vascular amyloid. (a) Double immunofluorescence of amyloid (Thio-S, green), and activated microglia (Clec7a, red) in leptomeninges of PDAPP mice treated with 3D6 or IgG control. Thio-S and Clec7a immunoreactivity overlay (Merge). (b) Double immunofluorescence of amyloid (Thio-S, green), and activated microglia (Clec7a, red) in penetrating vessels of PDAPP mice treated with 3D6 or IgG control. Thio-S and Clec7a immunoreactivity overlay (Merge). Scale bar 10 μm. [file 13024_2023_649_MOESM3_ESM.docx]

**
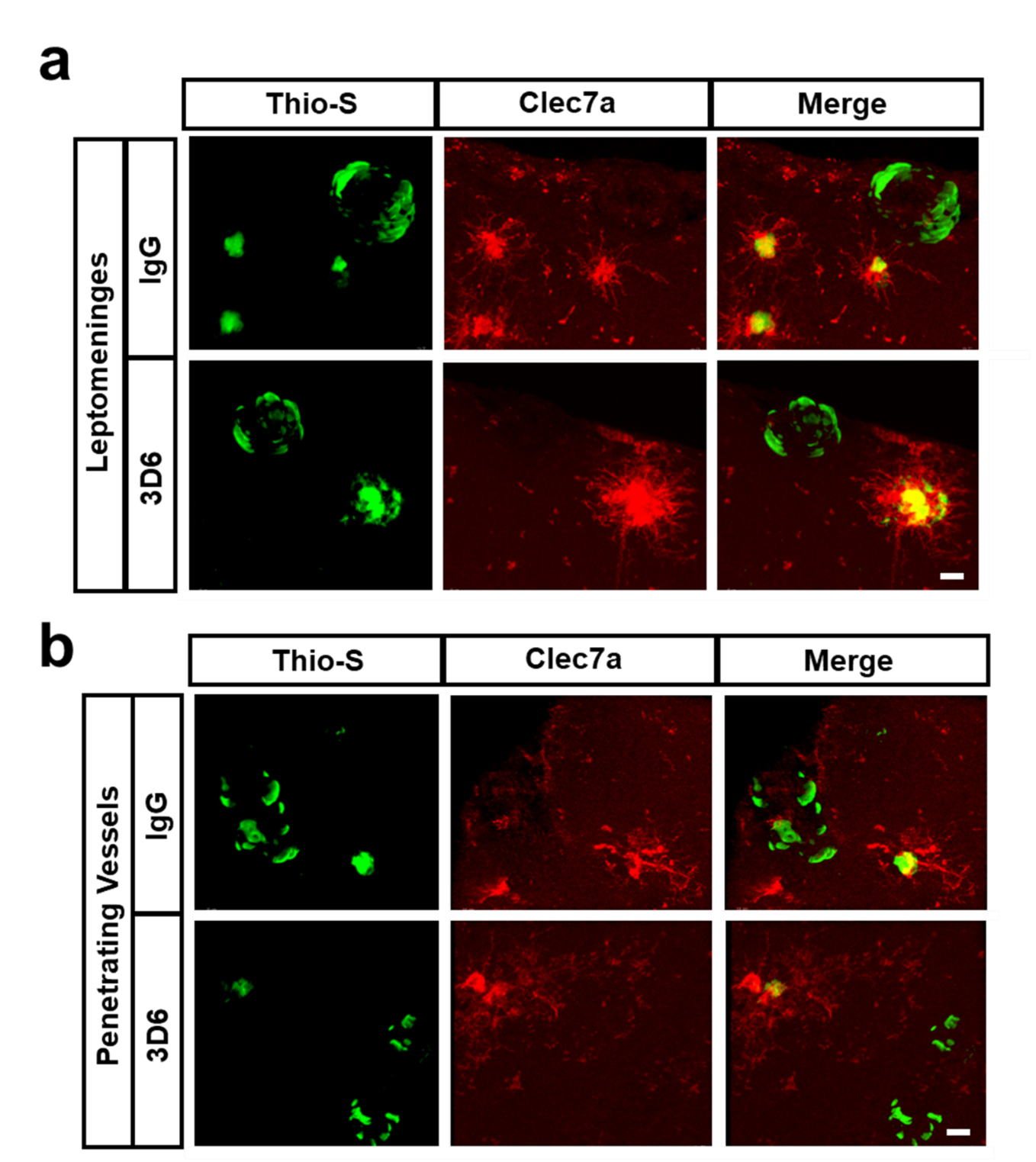
**

**Supplemental Figure 3. Clec7a^+^ microglia are not associated with vascular amyloid.** **(a)** Double immunofluorescence of amyloid (Thio-S, green), and activated microglia (Clec7a, red) in leptomeninges of PDAPP mice treated with 3D6 or IgG control. Thio-S and Clec7a immunoreactivity overlay (Merge). **(b)** Double immunofluocrescence of amyloid (Thio-S, green), and activated microglia (Clec7a, red) in penetrating vessels of PDAPP mice treated with 3D6 or IgG control. Thio-S and Clec7a immunoreactivity overlay (Merge). Scale bar 10 μm.
