# Supplementary material for: Amyloid-β (Aβ) immunotherapy induced microhemorrhages are associated with activated perivascular macrophages and peripheral monocyte recruitment in Alzheimer’s disease mice
Source: Mol Neurodegener. 2023 Aug 30;18:59. doi: 10.1186/s13024-023-00649-w (PMC10469415; doi:10.1186/s13024-023-00649-w)
Supplement: Supplementary file 4 — Supplemental Fig. 4: Perivascular macrophages exhibit enrichment exclusively with amyloid+ vessels in 3D6 treated PDAPP Mice. (a) Triple immunofluorescence of amyloid+ (Thio-S, green) or amyloid− vessels, perivascular macrophage (CD169, red) and endothelial cells (PECAM-1, cyan) in PDAPP mice treated with IgG control. Thio-S, CD169 and PECAM-1 immunoreactivity overlay (Merge). (b) Quantification of CD169+ area (%) of IgG in amyloid+ or amyloid− vessels. (c) Triple immunofluorescence of amyloid+ (Thio-S, green) or amyloid− vessels, perivascular macrophage (CD169, red) and endothelial cells (PECAM-1, cyan) in PDAPP mice treated with 3D6. Thio-S, CD169 and PECAM-1 immunoreactivity overlay (Merge). (d) Quantification of CD169+ area (%) of 3D6 in amyloid+ or amyloid− vessels. The number of vessels analyzed was 8–10 per animal. Results are shown as ± SEM of n = 6 (mice). Asterisks indicate significant differences, where ***p < 0.001 by unpaired Student’s t test. Scale bar 10 μm merge or 5 μm inset, respectively. [file 13024_2023_649_MOESM4_ESM.docx]

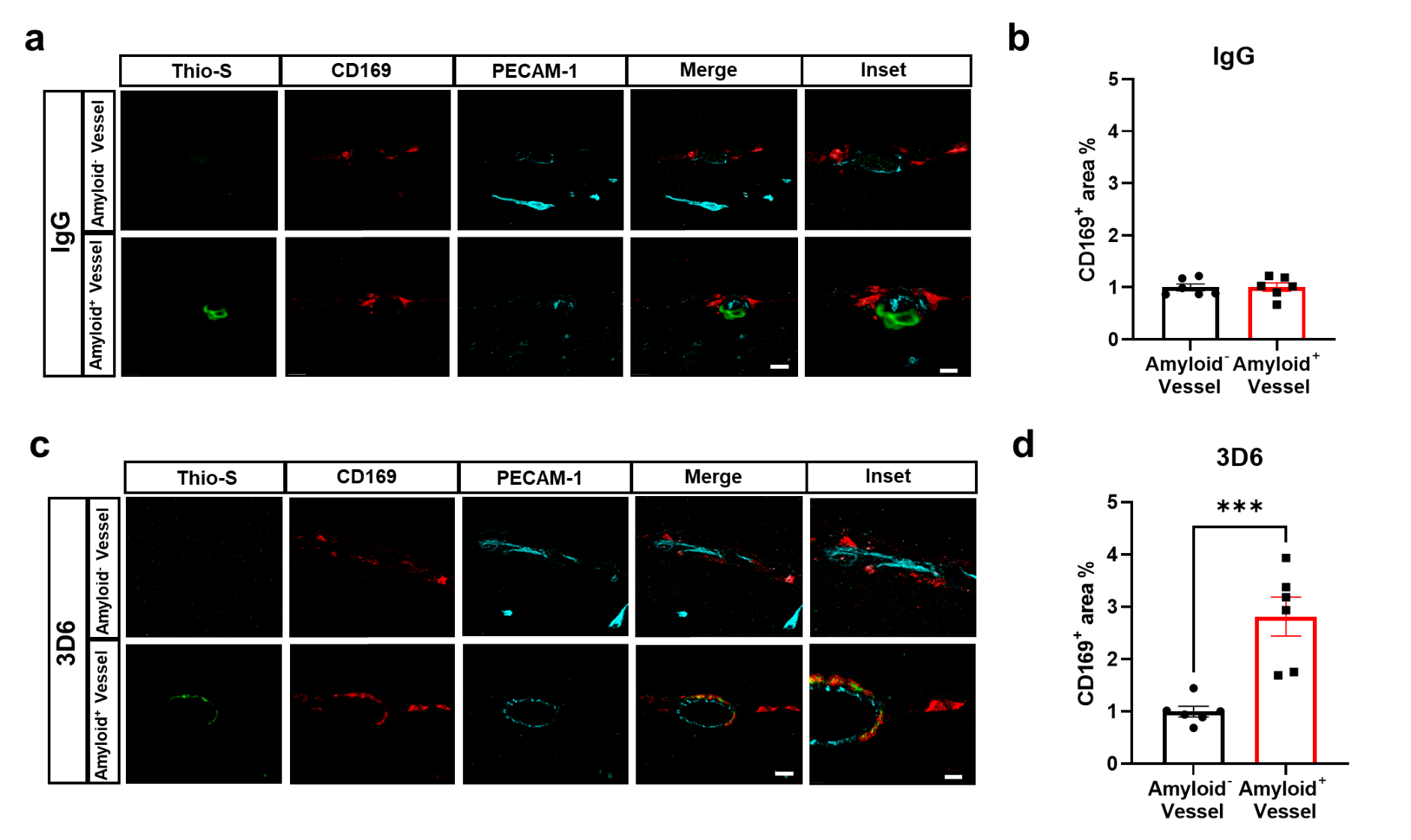


**Supplemental Figure 4. Perivascular macrophages exhibit enrichment exclusively with amyloid^+^ vessels in 3D6 treated PDAPP Mice**. (**a)** Triple immunofluorescence of amyloid^+^ (Thio-S, green) or amyloid^-^ vessels, perivascular macrophage (CD169, red) and endothelial cells (PECAM-1, cyan) in PDAPP mice treated with IgG control. Thio-S, CD169 and PECAM-1 immunoreactivity overlay (Merge). (**b)** Quantification of CD169^+^ area (%) of IgG in amyloid^+^ or amyloid^-^ vessels. (**c)**Triple immunofluorescence of amyloid^+^ (Thio-S, green) or amyloid^-^ vessels, perivascular macrophage (CD169, red) and endothelial cells (PECAM-1, cyan) in PDAPP mice treated with 3D6. Thio-S, CD169 and PECAM-1 immunoreactivity overlay (Merge). (**d)** Quantification of CD169^+^ area (%) of 3D6 in amyloid^+^ or amyloid^-^ vessels. The number of vessels analyzed was 8-10 per animal. Results are shown as ± SEM of n = 6 (mice). Asterisks indicate significant differences, where ****p* < 0.001 by unpaired Student's t test. Scale bar 10 μm merge or 5 μm inset, respectively.
