# Supplementary material for: Amyloid-β (Aβ) immunotherapy induced microhemorrhages are associated with activated perivascular macrophages and peripheral monocyte recruitment in Alzheimer’s disease mice
Source: Mol Neurodegener. 2023 Aug 30;18:59. doi: 10.1186/s13024-023-00649-w (PMC10469415; doi:10.1186/s13024-023-00649-w)
Supplement: Supplementary file 5 — Supplemental Fig. 5 Perivascular macrophages associated with vascular amyloid are CD206 and CD163 positive. Triple immunofluorescence of amyloid (X-34, blue), perivascular macrophages (CD206, red) (CD163, green) in PDAPP mice treated with 3D6 or IgG control. X-34, CD206 and CD163 immunoreactivity overlay (Merge). Scale bar 5 μm. [file 13024_2023_649_MOESM5_ESM.docx]

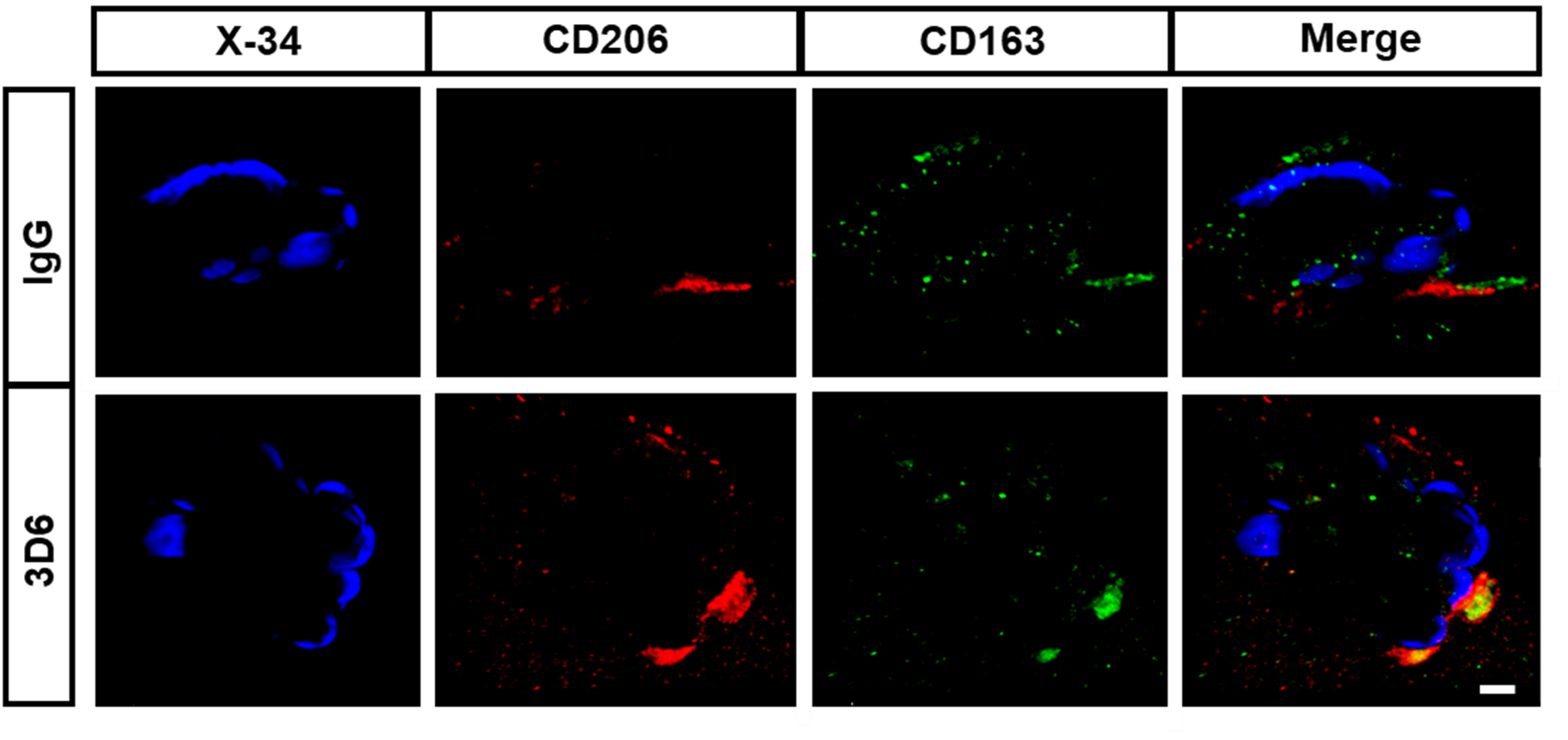


**Supplemental Figure 5. Perivascular macrophages associated with vascular amyloid are CD206 and CD163 positive.** Triple immunofluorescence of amyloid (X-34, blue), perivascular macrophages (CD206, red) (CD163, green) in PDAPP mice treated with 3D6 or IgG control. X-34, CD206 and CD163 immunoreactivity overlay (Merge). Scale bar 5 μm.
