# Supplementary material for: Amyloid-β (Aβ) immunotherapy induced microhemorrhages are associated with activated perivascular macrophages and peripheral monocyte recruitment in Alzheimer’s disease mice
Source: Mol Neurodegener. 2023 Aug 30;18:59. doi: 10.1186/s13024-023-00649-w (PMC10469415; doi:10.1186/s13024-023-00649-w)
Supplement: Supplementary file 6 — Supplemental Fig. 6 qPCR confirms dysregulated inflammatory signaling and extracellular matrix remodeling genes. (a) Relative expression of Pdpn in BMDM activated by IgG2a vs. control LALAPG (b) Relative expression of Timp1 in BMDM activated by IgG2a vs. control LALAPG (c) Relative expression of Msr1 in BMDM activated by IgG2a vs. control LALAPG. Data was normalized to the levels of actin mRNA. Relative quantitation was performed using 2−ΔΔCt (fold change) method. Results are shown as the mean ± SEM of n = 3 (independent cultures). Asterisks indicate significant differences, where * p < 0.05 and ** p < 0.01. [file 13024_2023_649_MOESM6_ESM.docx]

**
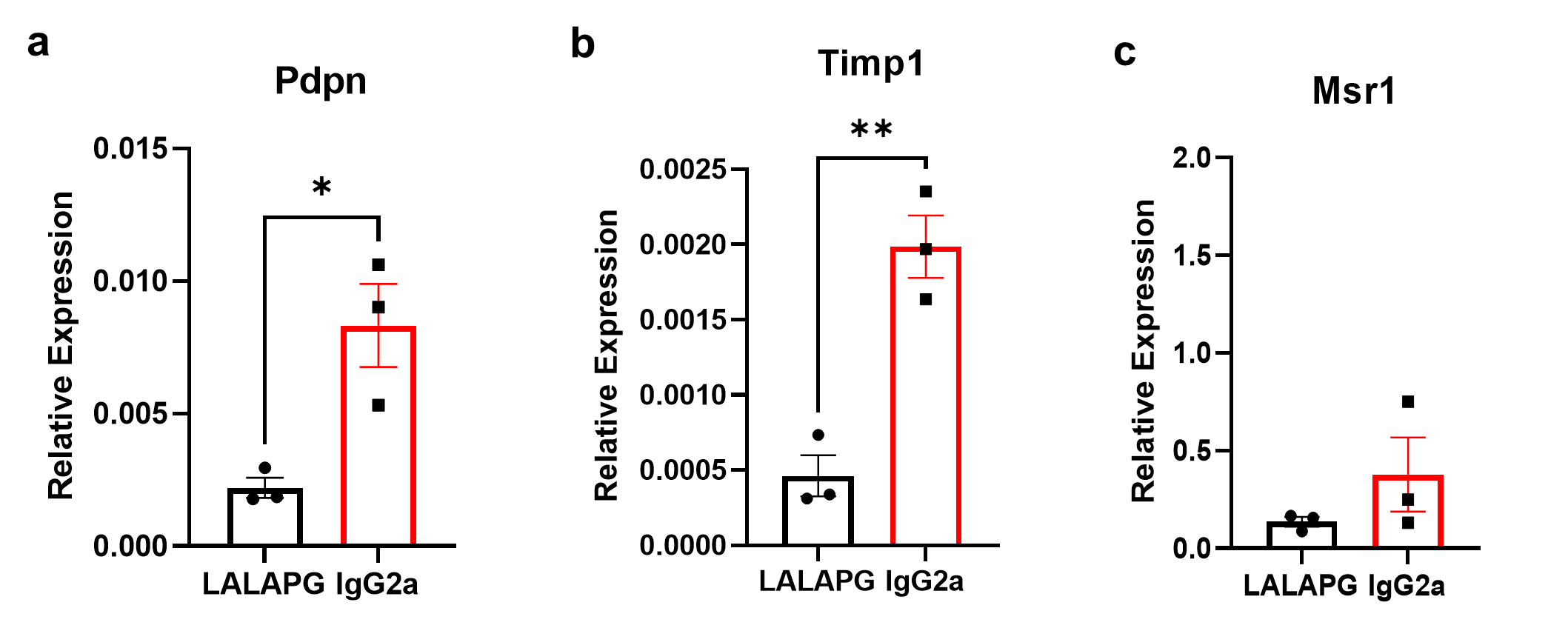
**

**Supplemental Figure 6. qPCR confirms dysregulated inflammatory signaling and extracellular matrix remodeling genes. a)** Relative expression of Pdpn in BMDM activated by IgG2a vs control LALAPG **b)** Relative expression of Timp1 in BMDM activated by IgG2a vs control LALAPG **c)** Relative expression of Msr1 in BMDM activated by IgG2a vs control LALAPG. Data was normalized to the levels of actin mRNA. Relative quantitation was performed using 2^-ΔΔCt^ (fold change) method. Results are shown as the mean ± SEM of n = 3 (independent cultures). Asterisks indicate significant differences, where * p < 0.05 and ** p < 0.01.
