# Supplementary material for: Amyloid-β (Aβ) immunotherapy induced microhemorrhages are associated with activated perivascular macrophages and peripheral monocyte recruitment in Alzheimer’s disease mice
Source: Mol Neurodegener. 2023 Aug 30;18:59. doi: 10.1186/s13024-023-00649-w (PMC10469415; doi:10.1186/s13024-023-00649-w)
Supplement: Supplementary file 7 — Supplemental Fig. 7 Bone marrow-derived macrophages plated with LALAPG have no significant differential gene expression from non-treatment group. (a) Schematic diagram showing macrophages treated with immunodeficient LALAPG control antibodies. (b) Volcano plots showing not significantly differentially expressed genes in LALAPG activated macrophages vs. control nontreated macrophages. Horizontal line values correspond to p-value < 0.01(solid), p-value < 0.05 (dashed). [file 13024_2023_649_MOESM7_ESM.docx]

**
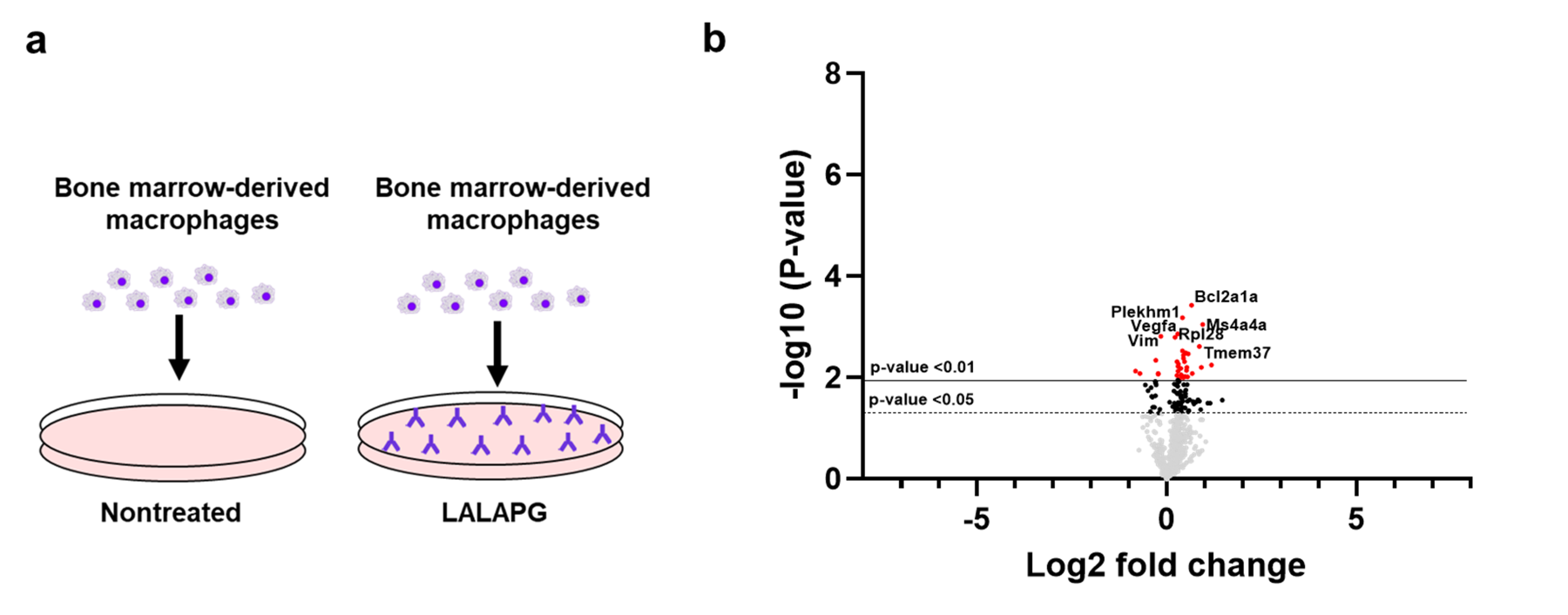
**

**Supplemental Figure 7. Bone marrow-derived macrophages plated with by LALAPG have no significant differential gene expression from non-treatment group. (a)** Schematic diagram showing macrophages treated with by immunodeficient LALAPG control antibodies. (**b)** Volcano plots showing not significantly differentially expressed genes in LALAPG activated macrophages vs. control nontreated macrophages. Horizontal line values correspond to p-value <.01(solid), p-value <.05(dashed).
