# Supplementary material for: Amyloid-β (Aβ) immunotherapy induced microhemorrhages are associated with activated perivascular macrophages and peripheral monocyte recruitment in Alzheimer’s disease mice
Source: Mol Neurodegener. 2023 Aug 30;18:59. doi: 10.1186/s13024-023-00649-w (PMC10469415; doi:10.1186/s13024-023-00649-w)
Supplement: Supplementary file 8 — Supplemental Fig. 8 Aβ immunotherapy activated perivascular macrophages of penetrating vessels express extracellular matrix remodeling genes. (a) Triple immunofluorescence of amyloid (Thio-S, green), Timp1 (red) and perivascular macrophages (CD169, cyan) in PDAPP mice treated with 3D6 or IgG control. Thio-S, Timp1 and CD169 immunoreactivity overlay (Merge). Colocalization analysis (white). (b) Quantification of Timp1+ area (%) of IgG or 3D6 treated mice. (c) Quantification of colocalization ratio of Timp1 and CD169. (d) Triple immunofluorescence of amyloid (Thio-S, green), MMP9 (red) and perivascular macrophages (CD169, cyan) in PDAPP mice treated with 3D6 or IgG control. Thio-S, MMP9 and CD169 immunoreactivity overlay (Merge). Colocalization analysis (white). (e) Quantification of MMP9+ area (%) of IgG or 3D6 treated mice. (f) Quantification of colocalization ratio of MMP9 and CD169. The number of vascular amyloid deposits analyzed was 8–10 per animal. Results are shown as ± SEM of n = 6 (mice). Asterisks indicate significant differences, where *p < 0.05 and ***p < 0.001 by unpaired Student’s t test. Scale bar 5 μm CC or 10 μm merge, respectively. [file 13024_2023_649_MOESM8_ESM.docx]

**
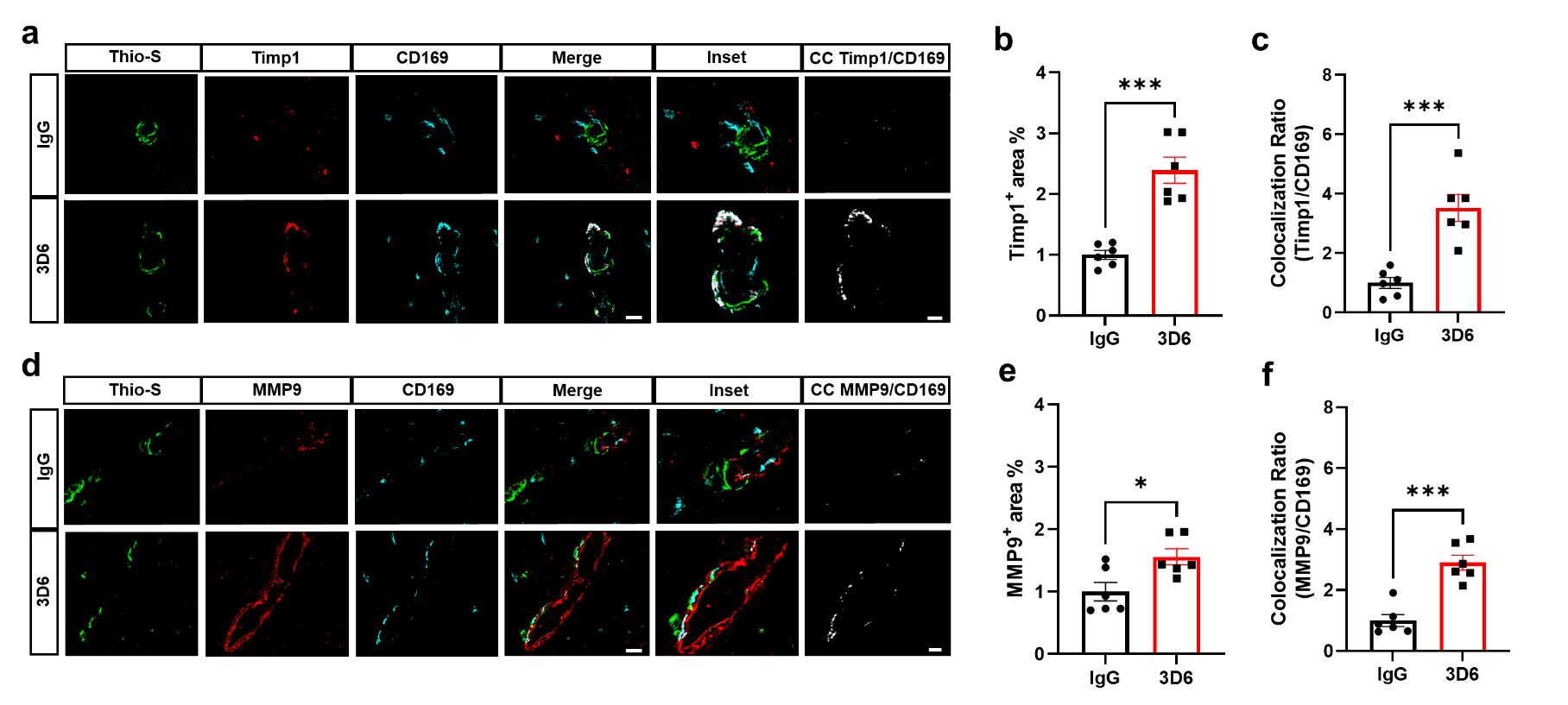
**

**Supplemental Figure 8. Aβ immunotherapy activated perivascular macrophages of penetrating vessels express extracellular matrix remodeling genes. (a****)** Triple immunofluorescence of amyloid (Thio-S, green), Timp1 (red) and perivascular macrophages (CD169, cyan) in PDAPP mice treated with 3D6 or IgG control. Thio-S, Timp1 and CD169 immunoreactivity overlay (Merge). Colocalization analysis (white). (**b)** Quantification of Timp1^+^ area (%) of IgG or 3D6 treated mice. (**c)** Quantification of colocalization ratio of Timp1 and CD169. (**d)** Triple immunofluorescence of amyloid (Thio-S, green), MMP9 (red) and perivascular macrophages (CD169, cyan) in PDAPP mice treated with 3D6 or IgG control. Thio-S, MMP9 and CD169 immunoreactivity overlay (Merge). Colocalization analysis (white). (**e)** Quantification of MMP9^+^ area (%) of IgG or 3D6 treated mice. (**f)** Quantification of colocalization ratio of MMP9 and CD169. The number of vascular amyloid deposits analyzed was 8-10 per animal. Results are shown as ± SEM of n = 6 (mice). Asterisks indicate significant differences, where *p<.05 and ****p* < 0.001 by unpaired Student's t test. Scale bar 5 μm CC or 10 μm merge, respectively.
