# Supplementary material for: Amyloid-β (Aβ) immunotherapy induced microhemorrhages are associated with activated perivascular macrophages and peripheral monocyte recruitment in Alzheimer’s disease mice
Source: Mol Neurodegener. 2023 Aug 30;18:59. doi: 10.1186/s13024-023-00649-w (PMC10469415; doi:10.1186/s13024-023-00649-w)
Supplement: Supplementary file 9 — Supplemental Fig. 9 Timp1+ immunoreactivity was not detected in astrocytes, microglia, or endothelial cells. (a) Four color immunofluorescence of amyloid (X-34, blue), astrocytes (GFAP, green), Timp1( red) and endothelial cells (PECAM-1) in PDAPP mice treated with 3D6 or IgG control. (b) Four color immunofluorescence of amyloid (X-34, blue), microglia (Clec7a, green), Timp1 (red) and endothelial cells (PECAM-1) in PDAPP mice treated with 3D6 or IgG control. Immunoreactivity overlay (Merge). Scale bar 10 μm. [file 13024_2023_649_MOESM9_ESM.docx]

**
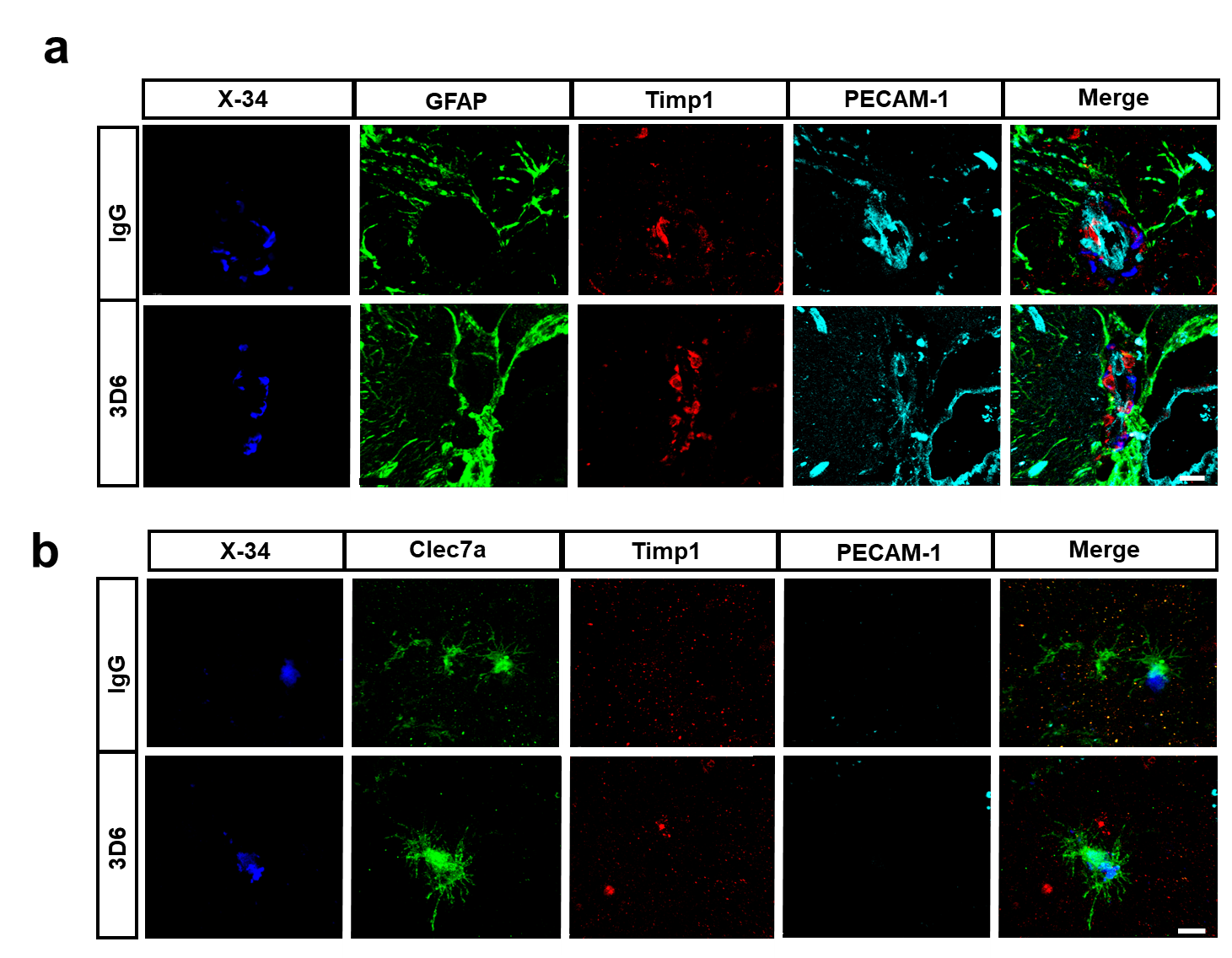
Supplemental Figure 9. Timp1^+^ immunoreactivity was not detected in astrocytes, microglia, or endothelial cells. (a)**  Four color immunofluorescence of amyloid (X-34, blue), astrocytes (GFAP, green), Timp1( red) and endothelial cells (PECAM-1) in PDAPP mice treated with 3D6 or IgG control. **(b)** Four color immunofluorescence of amyloid (X-34, blue), microglia (Clec7a, green), Timp1 (red) and endothelial cells (PECAM-1) in PDAPP mice treated with 3D6 or IgG control. Immunoreactivity overlay (Merge). Scale bar 10 μm.
