# Supplementary material for: Amyloid-β (Aβ) immunotherapy induced microhemorrhages are associated with activated perivascular macrophages and peripheral monocyte recruitment in Alzheimer’s disease mice
Source: Mol Neurodegener. 2023 Aug 30;18:59. doi: 10.1186/s13024-023-00649-w (PMC10469415; doi:10.1186/s13024-023-00649-w)
Supplement: Supplementary file 10 — Supplemental Fig. 10 Increased fibrinogen around vascular amyloid deposits in 3D6 treated PDAPP Mice. (a) Triple immunofluorescence of amyloid (Thio-S, green), fibrinogen (red) and endothelial cells (PECAM-1, cyan) in the leptomeninges of PDAPP mice treated with 3D6 or IgG control. Thio-S, fibrinogen, and endothelial cells (PECAM-1, cyan) immunoreactivity overlay (Merge). (b) Quantification of fibrinogen+ area (%) of IgG or 3D6 treated mice. (c) Triple immunofluorescence of amyloid (Thio-S, green), fibrinogen (red) and endothelial cells (PECAM-1, cyan) of penetrating vessels PDAPP mice treated with 3D6 or IgG control. Thio-S, fibrinogen, and PECAM-1 immunoreactivity overlay (Merge). (d) Quantification of fibrinogen+ area (%) of IgG or 3D6 treated mice. The number of vascular amyloid deposits analyzed was 8–10 per animal. Results are shown as ± SEM of n = 6 (mice). Asterisks indicate significant differences, where ** p < 0.01 and ***p < 0.001 by unpaired Student’s t test. Scale bar 5 μm merge or 10 μm inset, respectively. [file 13024_2023_649_MOESM10_ESM.docx]

**
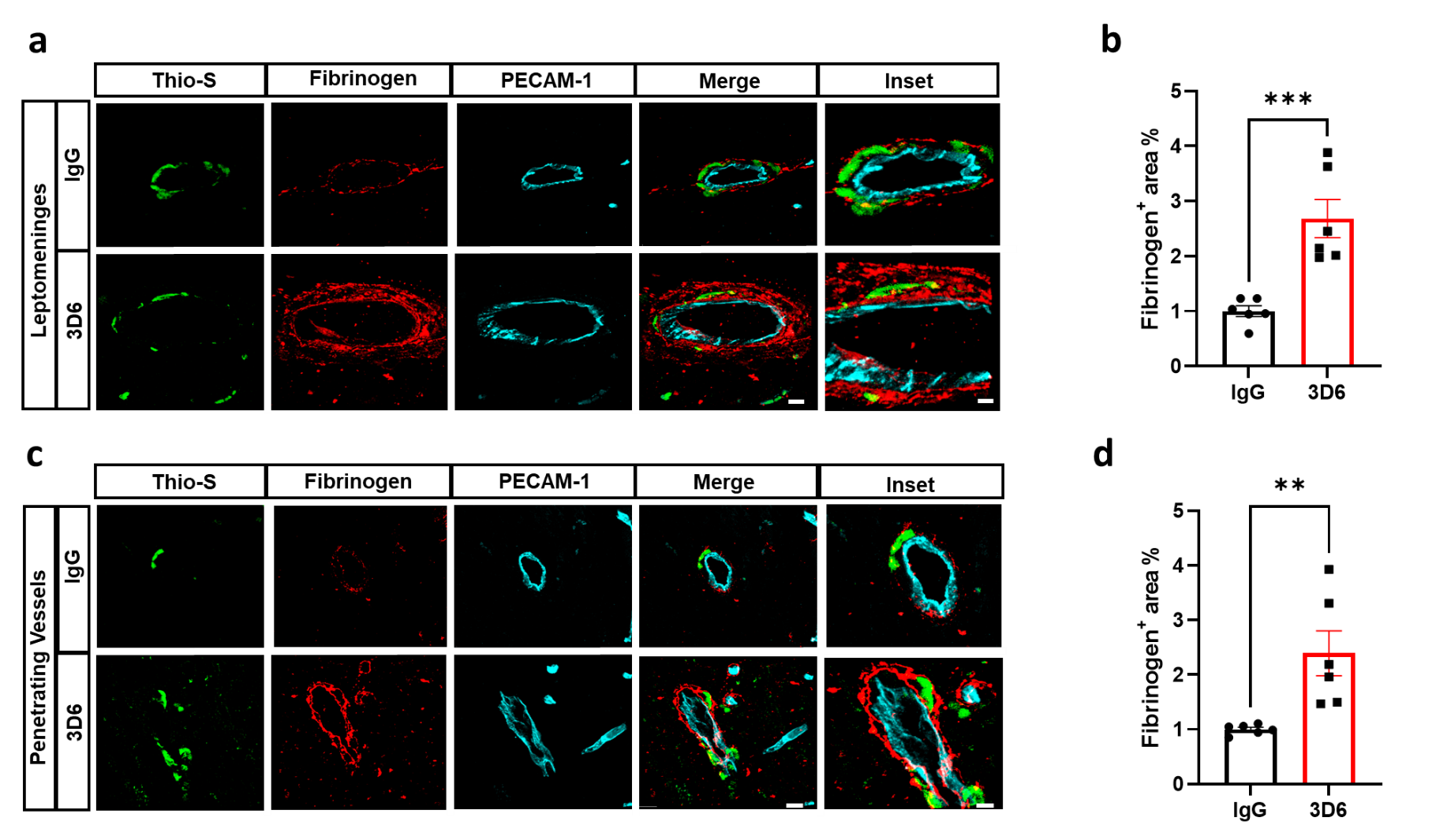
**

**Supplemental Figure 10. Increased fibrinogen around vascular amyloid deposits in 3D6 treated PDAPP Mice. (a)** Triple immunofluorescence of amyloid (Thio-S, green), fibrinogen (red) and endothelial cells (PECAM-1, cyan) in the leptomeninges of PDAPP mice treated with 3D6 or IgG control. Thio-S, fibrinogen, and endothelial cells (PECAM-1, cyan) immunoreactivity overlay (Merge). (**b)** Quantification of fibrinogen^+^ area (%) of IgG or 3D6 treated mice. (**c)** Triple immunofluorescence of amyloid (Thio-S, green), fibrinogen (red) and endothelial cells (PECAM-1, cyan) of penetrating vessels PDAPP mice treated with 3D6 or IgG control. Thio-S, fibrinogen, and PECAM-1 immunoreactivity overlay (Merge). (**d)** Quantification of fibrinogen^+^ area (%) of IgG or 3D6 treated mice. The number of vascular amyloid deposits analyzed was 8-10 per animal. Results are shown as ± SEM of n =6 (mice). Asterisks indicate significant differences, where ** p < .01 and ****p* < 0.001 by unpaired Student's t test. Scale bar 5 μm merge or 10 μm inset, respectively.
