# Supplementary material for: Amyloid-β (Aβ) immunotherapy induced microhemorrhages are associated with activated perivascular macrophages and peripheral monocyte recruitment in Alzheimer’s disease mice
Source: Mol Neurodegener. 2023 Aug 30;18:59. doi: 10.1186/s13024-023-00649-w (PMC10469415; doi:10.1186/s13024-023-00649-w)
Supplement: Supplementary file 11 — Supplemental Fig. 11 Macrophages activated by Fc receptor signaling have biological processes and molecular functions enriched in chemotactic signaling. (a,b) DAVID bioinformatics gene ontology enrichment analysis of differentially expressed genes upregulated in bone marrow-derived macrophages activated by Fc receptor signaling. [file 13024_2023_649_MOESM11_ESM.docx]

**
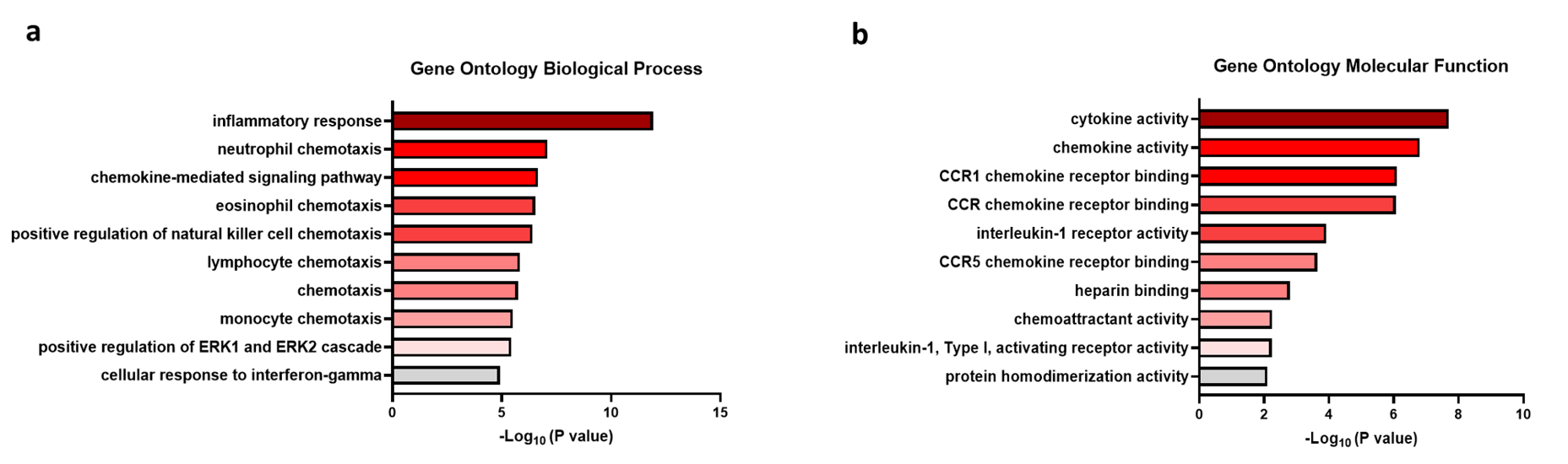
**

**Supplemental Figure 11. Macrophages activated by Fc receptor signaling have biological processes and molecular functions enriched in chemotactic signaling.** (**a,b)** DAVID bioinformatics gene ontology enrichment analysis of differentially expressed genes upregulated in bone marrow-derived macrophages activated by Fc receptor signaling.
